# Temporal trends in pregnancy outcomes during a health system shock

**DOI:** 10.1038/s43856-026-01493-x

**Published:** 2026-05-07

**Authors:** Florence Tydeman, Kathryn V. Dalrymple, Alice McGreevy, Lucilla Poston, Tisha Dasgupta, Abigail Easter, Asma Khalil, Lisa Long, Hiten D. Mistry, Daghni Rajasingam, Sergio A. Silverio, Marina Soley-Bori, Yanzhong Wang, Sara L. White, The RESILIENT Study Group, Lucilla Poston, Abigail Easter, Asma Khalil, Sergio A. Silverio, Marina Soley-Bori, Sara L. White, Debra Bick, Harriet Boulding, Emma L. Duncan, Julia Fox-Rushby, Hiten D. Mistry, Eugene C. Nelson, Paul Seed, Aricca D. Van Citters, Ingrid Wolfe, Peter von Dadelszen, Laura A. Magee, The Early Life Cross Linkage in Research, Born in South London (eLIXIR-BiSL) Partnership, Lucilla Poston, Tisha Dasgupta, Ingrid Wolfe, Robert Stewart, David Edwards, Mark Ashworth, Jane Sandall, Cheryl Gillett, Michael Absoud, Lucy Pickard, Amanda Grey, Sarah Spring, Toyin Kazeem, Amelia Jewell, Matthew Broadbent, Finola Higgins, Leonardo de Jongh, Carolyn Gill, Laura A. Magee, Peter von Dadelszen, Laura A. Magee

**Affiliations:** 1 Unit for Medical Statistics, School of Life Course & Population Health Sciences, King’s College London, London, UK; 2 Department of Women & Children’s Health, School of Life Course & Population Health Sciences, King’s College London, London, UK; 3 Department of Nutritional Sciences, School of Life Course & Population Health Sciences, King’s College London, London, UK; 4 Department of Population Health Sciences, School of Life Course & Population Health Sciences, King’s College London, London, UK; 5 Fetal Medicine Unit, Department of Obstetrics and Gynaecology, St. George’s University Hospitals NHS Foundation Trust, London, UK; 6 Fetal Medicine Unit, Department of Obstetrics and Gynaecology, Liverpool Women’s NHS Foundation Trust, Liverpool, UK; 7 Department of Obstetrics and Gynaecology, King’s College Hospital NHS Foundation Trust, London, UK; 8 Department of Population Health Sciences, College of Life Sciences, University of Leicester, Leicester, UK; 9 Department of Obstetrics and Gynaecology, Guy’s and St. Thomas’ NHS Foundation Trust, London, UK; 10 Department of Psychology, Institute of Population Health, University of Liverpool, Liverpool, UK; 11 Department of Diabetes and Endocrinology, Guy’s and St Thomas’ Hospitals NHS Foundation Trust, London, UK; 12 Warwick Clinical Trials Unit, University of Warwick, Coventry, UK; 13 The Policy Institute at King’s, King’s College London, London, UK; 14 Department of Twin Research and Genetic Epidemiology, King’s College London, London, UK; 15 The Geisel School of Medicine at Dartmouth, The Dartmouth Institute for Health Policy and Clinical Practice, Dartmouth, US; 16 Department of Psychological Medicine, Institute of Psychiatry, Psychology and Neuroscience, King’s College London and NIHR Maudsley Biomedical Research Centre, South London and Maudsley NHS Foundation Trust, London, UK; 17 Department of Perinatal Imaging and Health, King’s College London, London, UK; 18 Department of Comprehensive Cancer Centre, School of Cancer & Pharmaceutical Sciences, King’s College London, London, UK; 19 Evelina London Children’s Healthcare, London, UK; 20 King’s College Hospital NHS Foundation Trust, London, UK; 21 eLIXIR-BiSL Oversight Committee, King’s College London, London, UK; 22 South London and Maudsley NHS Foundation Trust, London, UK; 23 NIHR Maudsley Biomedical Research Centre, South London and Maudsley NHS Foundation Trust, London, UK; 24 Guy’s and St Thomas’ NHS Foundation Trust, London, UK; 25 School of Life Course and Population Sciences, King’s College London, London, UK

**Keywords:** Epidemiology, Epidemiology

## Abstract

**Background:**

To better understand reported COVID-19 pandemic effects on pregnancy, we examined temporal trends in pregnancy outcomes in a diverse population from South London, United Kingdom.

**Methods:**

We included 31,411 singleton pregnancies with complete registration and birth outcomes across pre-pandemic (May 1, 2019–March 22, 2020, 24.5%), pandemic lockdowns (March 23, 2020–July 17, 2021, 32.3%), and pandemic without lockdown epochs (July 18, 2021–April 22, 2023, 43.2%). Multivariable regression was employed to evaluate outcomes by study epoch, adjusting for potential confounders (e.g., ethnicity, deprivation, site), followed by generalized additive modelling to visualise monthly trends.

**Results:**

Here we show that of 17 outcomes: six have stable trends (e.g., preterm birth, stillbirth); eight show linear trends, either decreasing (e.g., gestational age at birth, vaginal tears) or increasing (e.g., Caesareans, postpartum haemorrhage); and three show quadratic (complex) trends (e.g., secondary mental health services, labour induction).

**Conclusions:**

Overall, most outcomes during the pandemic mirror pre-pandemic trends, with fluctuations observed likely due to site-specific responses. Our findings highlight the need for temporal analysis of pregnancy outcomes.

## Plain Language Summary

We studied how the COVID-19 pandemic affected pregnancy outcomes in South London. We analysed more than 31,000 pregnancies from before the pandemic, during lockdowns, and after restrictions were lifted. We compared outcomes across these periods while accounting for factors like ethnicity, deprivation, and hospital site. Of 17 outcomes, six (such as stillbirth) stayed stable over time. Eight outcomes showed steady changes: some decreased (like vaginal tears), while others increased (such as Caesarean births). Three outcomes (including referrals to mental health services) showed complex patterns. Overall, most changes during the pandemic continued earlier trends rather than being caused by the pandemic itself. Some differences may reflect how individual hospitals responded. These results highlight the importance of looking at outcomes over time.

## Abstract (web summary)

Tydeman et al. analyze pregnancy outcomes before the COVID-19 pandemic, during lockdowns, and after restrictions were lifted in >30,000 pregnancies from ethnically and socio-economically diverse individuals in the UK. Most pregnancy outcomes follow pre-pandemic temporal trends, either stable, increasing, or decreasing.

## Introduction

During the COVID-19 pandemic, pregnant women and birthing people were at risk from direct effects of SARS-CoV-2 infection and indirect effects of maternity service reconfigurations. Whilst a small proportion ( ≈ 9%) of pregnant or recently pregnant women attending hospital were diagnosed with suspected or confirmed COVID-19 infection,^1^ all women accessing maternity care were affected by maternity care changes, alongside societal changes in social interaction, isolation, and activity.^2^ Maternity service reconfigurations included a shift to provision of at least some care via telephone or videoconference; a reduction in the number of antenatal visits; and an increase in self-monitoring for pregnancy-related complications, such as hypertension.^3^

Globally, the impact of the pandemic on pregnancy outcomes has been examined primarily in the first pandemic year or slightly beyond into the summer of 2021.^4,5^ Many studies have found the pandemic was associated with a reduction in: preterm birth (particularly spontaneous preterm birth), and small-for-gestational age (SGA) babies; increases in mental health problems^6^ and intervention at birth, such as labour induction and Caesarean birth;^4,5^ and no effect on stillbirth or neonatal death.^7^ However, others have reported a lower incidence of labour induction for fetal growth restriction (FGR), in the absence of commonly-associated adverse outcomes.^8^ Pandemic effects on outcomes have varied by ethnicity, but not social deprivation.^4^

To inform post-pandemic maternity care planning, this study extends the period of observation in other studies, and evaluates trends across pre-pandemic, pandemic lockdowns, and pandemic without lockdown phases, in key indicators measured at birth in the UK. These reflect organisational performance, clinical quality improvement, and national maternity outcomes. Of 17 outcomes: six have stable trends, eight show linear trends, either decreasing or increasing; and three show complex trends. Overall, most outcomes during the pandemic mirror pre-pandemic trends, with fluctuations likely due to site-specific responses, rather than the pandemic itself.

## Methods

### The Early Life Cross Linkage in Research—Born in South London data linkage

Data for this retrospective longitudinal study were obtained from the ‘eLIXIR (Early Life Cross Linkage in Research)-Born in South London (BiSL)’ data linkage, the methods for which have been published previously.^9^ In brief, these data are routinely-collected, linked maternity, neonatal, and maternal mental health records from two maternity hospitals (Guy’s and St. Thomas’ NHS Foundation Trust, and King’s College Hospital NHS Foundation Trust), in South London, United Kingdom (UK), and South London and Maudsley NHS Foundation Trust (SLaM). Both maternity hospitals provide services that cover community-based, standard-risk, as well as high-risk specialist care to ethnically- and socioeconomically-diverse populations. In line with recommendations from the Royal College of Obstetricians and Gynaecologists and Royal College of Midwives, like most UK maternity units, GSTT and KCH: reduced antenatal contacts, some of which (particularly in early pregnancy) were converted to remote consultations, increased use of self-monitoring of blood pressure, modified screening for gestational diabetes, reduced the frequency of fetal growth surveillance by ultrasound, and reduced options for place of birth.^19^ Of note, there were few changes to labour induction indications or the offer of Caesarean by request. Site-specific differences were that GSTT, which has a greater focus on maternal high-risk pregnancies, maintained OGTT testing for women at increased risk of GDM. KCH as a centre of excellence for fetal medicine and pre-eclampsia screening, maintained these services, from 11−13 weeks’ gestation; this centre is also co-located with perinatal mental health expertise.

The eLIXIR-BiSL dataset includes pseudonymised, structured and comprehensive records from antenatal booking to postnatal follow-up, covering ~15,000 births annually in an ethnically and socio-demographically diverse population of South London, UK. Inclusion is based on opt-out consent.

Raw clinical data are extracted from BadgerNet Systems, and then sent securely to the staff at the SLaM Clinical Data Linkage Service and within their Trusted Research Environment, where they are linked using their common identifiers, with mental health data (as relevant) from the Case Record Interactive Service system at SLaM,^10^ and primary care records from Lambeth DataNet. Match quality is 98.5% for infants with their mothers’ records within BadgerNet. Duplicate records are excluded, and identified as those related to transfer of care and registration at two hospitals, identified based on the presence of two or more antenatal care registration IDs with estimated delivery dates within 14 days; only the first record is kept. Results from all linkages (current and prior) are stored within the Clinical Data Linkage Service [CDLS] ‘data safe haven’. The process is summarised in Supplementary Fig. 1. As such, the data are available in an identifiable format to only a small number of data-processing staff, in accordance with data sharing contracts between the data provider institutions. Operational management, in accordance with the eLIXIR-BiSL Security Model, is provided by the eLIXIR Oversight Committee. The data linkage platform has been approved as an anonymised dataset for medical research (Oxford Research Ethics Committee C, ref. /SC/0086, 2018-23; renewal 23/SC/0116, 2023-8). The data are not coded, but we have previously replicated findings in external, comparable data resources.^11^ Inclusion is based on opt-out consent.

Applications to use eLIXIR-BiSL data are made on a research application form, reviewed by the eLIXIR-BiSL Oversight Committee. Following approval, the researcher is provided with controlled user access to the project-specific de-identified data extract, provided by the ClDS staff and placed on the eLIXIR-BiSL secure server, partitioned from the Clinical Data Linkage Service ‘data safe haven’ where the identifiers are located.

### Participants

The target population was women and birthing people who: (i) did not opt-out nationally from research or locally from eLIXIR-BiSL data linkage (as the current course of action by ≈0.03% of women); (ii) registered for antenatal care at the relevant NHS hospital (site, as above), between 01/October/2018 (when data linkage began) and 30/April/2023 (the date up to which data were available for both sites) (*N* = 54,924); and (iii) had both antenatal registration and birth records and dates (*N* = 36,985) (Supplementary Fig. 2).

We included only one birth per woman and/or birthing person. Those with more than one birth during the study period had one pregnancy randomly included within the data, using the method of Langham et al.,^12^ allowing simplification of the data structure. (See Supplementary Table 1 for a comparison of patient characteristics between the full and reduced data, excluding multiple pregnancies per woman.)

We excluded multifetal pregnancies, as is commonly done due to their high-risk nature, and to support a simplified data structure for birth outcomes.^13^

We truncated the data to exclude pregnancies outside the timeframe of 01 May 2019, to 22 April 2023 (Supplementary Fig. 3), as linkage across antenatal bookings and delivery data showed tails when summarising the total number of deliveries and/or bookings per month. This was due to pregnancies with late antenatal registration in the antenatal booking data, and preterm births in the delivery data. We chose to truncate the data for a stable ‘number of deliveries’ denominator, to avoid biasing our data for records at the beginning and end of the study period.

### Data collection and outcomes

Baseline maternal and pregnancy characteristics at antenatal registration were demographic factors, and current and past obstetric, medical, social (including socio-economic status), and mental health history. All variable are listed fully in Table 1, and include the following indicators of organisation performance measured at antenatal registration: maternal age, ethnicity, index of multiple deprivation (IMD),^14^ prior Caesarean, and gestational age at booking. Ethnicity was defined using the eight-category classification by the Office for National Statistics, UK: White, Black/African/Caribbean/Black British, Indian (Asian or Asian British), Pakistani (Asian or Asian British), Other Asian/Asian British, Mixed/multiple ethnic groups, Any other ethnic group, or Not stated/unknown.^15^ The Index of Multiple Deprivation (IMD) was used as the standard measure in the UK; IMD uses postal code to give an overall measure of deprivation within an defined geographic area (roughly equivalent to a neighbourhood of 1000–3000 people), and incorporates the domains of: income, employment, educations skills and training, health deprivation and disability, crime, barriers to housing and services, and living environment.^14^

**Table 1:** Baseline participant pregnancy characteristics, and pregnancy outcomes

	Overall	Pre-pandemic	Pandemic with lockdowns	Pandemic without lockdowns
Antenatal booking	*N* = 31,411^a^	*N* = 7706^a^	*N* = 10,137^a^	*N* = 13,568^a^
Data source				
Site A	18,728 (59.6%)	4471 (58%)	5949 (59%)	8308 (61%)
Site B	12,683 (40.4%)	3235 (42%)	4188 (41%)	5260 (39%)
Gestation at booking (weeks)	9.00 (8.00, 12.00)	10.00 (8.00, 12.00)	9.00 (8.00, 12.00)	10.00 (8.00, 12.00)
Ethnicity				
White	15,887 (51%)	3812 (49%)	5338 (53%)	6737 (50%)
Black/African/Caribbean/Black British	6309 (20%)	1442 (19%)	2063 (20%)	2804 (21%)
Indian (Asian or Asian British)	765 (2.4%)	160 (2.1%)	241 (2.4%)	364 (2.7%)
Mixed/multiple ethnic groups	1636 (5.2%)	326 (4.2%)	529 (5.2%)	781 (5.8%)
Other Asian/Asian British	1930 (6.1%)	386 (5.0%)	627 (6.2%)	917 (6.8%)
Pakistani (Asian or Asian British)	327 (1.0%)	72 (0.9%)	111 (1.1%)	144 (1.1%)
Any other ethnic group	2186 (7.0%)	499 (6.5%)	795 (7.8%)	892 (6.6%)
(Missing)	2371 (7.5%)	1009 (13%)	433 (4.3%)	929 (6.8%)
IMD quintile				
1 (most deprived)	6050 (19%)	1460 (19%)	1900 (19%)	2690 (20%)
2	12,914 (41%)	3230 (42%)	4167 (41%)	5517 (41%)
3	7790 (25%)	1979 (26%)	2563 (25%)	3248 (24%)
4	2906 (9.3%)	685 (8.9%)	932 (9.2%)	1289 (9.5%)
5 (least deprived)	1210 (3.9%)	267 (3.5%)	400 (3.9%)	543 (4.0%)
(Missing)	541 (1.7%)	85 (1.1%)	175 (1.7%)	281 (2.1%)
Nulliparous	17,226 (55%)	3922 (51%)	5459 (54%)	7845 (58%)
Smoker at booking	1126 (3.6%)	301 (3.9%)	357 (3.5%)	468 (3.4%)
Previous Caesarean	4475 (14%)	1139 (15%)	1459 (14%)	1877 (14%)
Pregnancy outcomes				
Gestation at birth (Wks)	39.00 (38.00, 40.00)	39.00 (38.00, 40.00)	39.00 (38.00, 40.00)	39.00 (38.00, 40.00)
Smoker at birth	825 (2.6%)	216 (2.8%)	278 (2.7%)	331 (2.4%)
Mental health service accessed				
Any (includes inpatient)	2286 (7.3%)	532 (6.9%)	651 (6.4%)	1103 (8.1%)
NHS ‘Talking Therapies’^b^	1372 (4.4%)	297 (3.9%)	420 (4.1%)	655 (4.8%)
‘Community contacts’^c^	1176 (3.7%)	285 (3.7%)	298 (2.9%)	593 (4.4%)
Preterm birth	2074 (6.6%)	535 (6.9%)	634 (6.3%)	905 (6.7%)
Induction of labour	6845 (22%)	1430 (19%)	2323 (23%)	3092 (23%)
Unassisted vaginal birth	14,368 (46%)	3862 (50%)	4772 (47%)	5734 (42%)
(Missing)	84 (0.3%)	8 (0.1%)	28 (0.3%)	48 (0.4%)
Interventional delivery				
Emergency Caesarean	7062 (22%)	1473 (19%)	2123 (21%)	3466 (26%)
(Missing)	84 (0.3%)	8 (0.1%)	28 (0.3%)	48 (0.4%)
Elective Caesarean	5064 (16%)	1115 (14%)	1580 (16%)	2369 (17%)
(Missing)	84 (0.3%)	8 (0.1%)	28 (0.3%)	48 (0.4%)
Assisted vaginal birth	4833 (15%)	1248 (16%)	1634 (16%)	1951 (14%)
(Missing)	84 (0.3%)	8 (0.1%)	28 (0.3%)	48 (0.4%)
Vaginal tear (3^rd^/4^th^)	500 (1.6%)	146 (1.9%)	144 (1.4%)	210 (1.5%)
PPH	3186 (10%)	731 (9.5%)	976 (9.6%)	1479 (11%)
(Missing)	1 ( < 0.1%)	0 (0%)	0 (0%)	1 ( < 0.1%)
Stillbirth	163 (0.5%)	35 (0.5%)	55 (0.5%)	73 (0.5%)
5-min Apgar <7	502 (1.6%)	121 (1.6%)	157 (1.5%)	224 (1.7%)
(Missing)	934 (3.0%)	202 (2.6%)	298 (2.9%)	434 (3.2%)
SGA	2242 (7.1%)	546 (7.1%)	681 (6.7%)	1015 (7.5%)
(Missing)	75 (0.2%)	19 (0.2%)	16 (0.2%)	40 (0.3%)
LGA	3841 (12%)	959 (12%)	1270 (13%)	1612 (12%)
(Missing)	75 (0.2%)	19 (0.2%)	16 (0.2%)	40 (0.3%)
Admitted to NICU	2072 (6.6%)	576 (7.5%)	737 (7.3%)	759 (5.6%)

*ICU* intensive care unit, *IMD* Index of multiple deprivation, *IQR* interquartile range, *LGA* large-for-gestational age, *NHS* National Health Service, *NICU* neonatal intensive care unit, *PPH* postpartum haemorrhage, *SGA* small-for-gestational age, *Wk* weeks

^a^*n* (%); Median (IQR),

^b^NHS ‘Talking Therapies’ are psychological therapies for anxiety and depression (https://www.england.nhs.uk/mental-health/adults/nhs-talking-therapies/).

^c^These are secondary mental health services community consultations

We evaluated trends in key pregnancy and delivery indicators across three epochs, according to delivery date: pre-pandemic (01 October 2018 to 22 March 2020), pandemic lockdowns (23 March 2020 to 17 July 2021), and pandemic without lockdown (18 July 2021 to 4 May 2023).^16^ Key indicators measured at birth reflected UK: (i) Organisational Performance Indicators (i.e., interventional birth, defined as Caesarean birth or operative vaginal delivery); (ii) Clinical Quality Improvement Metrics (i.e., smoker at delivery, gestational age at birth, preterm birth <37 weeks, mode of birth, 3rd or 4th degree vaginal tears, and 5-minute Apgar <7); and (iii) National Maternity Indicators (i.e., mode of birth, 3rd or 4th degree tears, postpartum haemorrhage [PPH], 5-minute Apgar <7, birthweight <10th centile.^17^ Additionally, we included other standard pregnancy outcomes (i.e., labour induction, stillbirth, large-for-gestational age [LGA, >90th centile] infants, and neonatal care unit [NICU] admission), and mental health outcomes given particular interest in these related to the pandemic (i.e., maternal utilisation of mental health services, as NHS ‘Talking Therapies’ [psychological therapies for anxiety and depression, developed to improve access and delivery of relevant treatment and previously known as Improving Access to Psychological Therapies^18^], secondary mental health services ‘community contacts’, and mental health-related inpatient admissions).

### Statistics and reproducibility

Descriptive statistics allowed for presentation of data, overall and by pandemic period, as mean (SD), median (IQR), and counts (percentage), as appropriate by data type and normality assumptions.

The primary analysis compared the incidence of each maternal outcome across pandemic phases (as above^6^), using multivariable regression. Ordinal factor trend testing was employed by treating pandemic phases as a three-stage ordinal predictor variable and tested for evidence of flat (no trend), linear, and/or quadratic relationships over time. Adjustment factors were site (A or B) and variables based on published literature: ethnicity, IMD quintile, gestational age at booking (weeks), smoking at booking, nulliparity, prior Caesarean and month. A dummy variable for month was included in all models, to account for seasonality. The overall contribution was assessed using an ANOVA *F*-test, and the global *p*-value was reported to evaluate seasonality. BMI was not included as an adjustment factor due to the significant increase in its missingness during pandemic lockdowns, related to less face-to-face care; inclusion of BMI could have introduced bias due to non-random missingness.

Regression estimates, or odd ratios (ORs), with 95% confidence intervals (CIs) were reported for each delivery outcome for pandemic vs pandemic lockdowns, and pandemic lockdowns vs pandemic without lockdown, and additionally for each covariate. Interactions for each delivery outcome were tested between pandemic phase and: site, ethnicity, and IMD, to examine whether the pandemic exacerbated inequalities.

A secondary analysis visualised temporal trends in maternity outcomes in more granularity, by month, using generalised additive models (GAMs). These allow modelling and visualisation of complex and non-linear relationships. Given the timing and intensity of pandemic-related interventions, GAMs provided an appropriately flexible framework to capture the potential effects on outcomes of gradual and variable changes in care patterns, particularly at an individual level. For each delivery outcome, GAMs were plotted to illustrate trends, using flexible thin-plate regression splines stratified by site, from 1 May 2019 to 22 April 2023. The data were interpreted in terms of predicted means or probabilities of delivery outcomes, contingent upon the outcome distribution, per month/year of the study period. All outcomes were adjusted for the same confounders, as above.

A sensitivity analysis was conducted across all outcomes to assess the impact of time spent in pandemic epoch “pandemic with lockdowns”, per trimester. This was calculated by the proportion of days within each trimester within the defined “pandemic with lockdowns” period between March 2020 and July 2021. Multivariable regression analyses, adjusting for the same covariates as in the primary analysis was used to assess the relationship between maternal and birth outcome and the proportion of time spent in a lockdown period across three trimester variables, to provide an indication of the lockdown effect during the antenatal period, and not solely by delivery date.

All analyses and data cleaning procedures were executed using RStudio version 4.3.0. A two-sided significance level of 1% was considered throughout, to account for the number of analyses and the large sample size. Missing data were reported in descriptive analyses and modelled as their own category throughout, providing insights into missingness. This was a convenience sample of available data, so there was no sample size calculation.

## Results

Of 36,985 potentially eligible pregnancies, excluded were: 727 multifetal gestations, a random 3298 pregnancies to mothers with more than one pregnancy, and 1549 pregnancies during truncation (Supplementary Fig. 2). As a result, there were 31,411 pregnancies included in our analysis, from sites A (59.6%) and Site B (40.4%), over pre-pandemic (*N* = 7706; 24.5%), pandemic lockdowns (*N* = 10,137; 32.3%), and pandemic without lockdown epochs (*N* = 13,568; 43.2%) (Table 1).

### Baseline characteristics

Throughout the study period, maternal and pregnancy characteristics remained similar (Table 1). The gestation at booking was 9−10 weeks. Approximately half of the cohort was from a minority ethnic group, primarily Black ethnicity. Just over half of pregnancies were from the two most socially-deprived IMD quintiles. Just over half of women were nulliparous, few reported smoking at antenatal booking, and about one-sixth had undergone prior Caesarean.

### Outcomes over time

Across study epochs, there were differences in many of the maternal outcomes evaluated (Table 1). Median gestational age at birth (39 weeks [IQR 38−40]) and smoking at birth (just under 3%) remained steady over time. However, there appeared to be a progressive rise in use of NHS Talking Therapies, and an initial decrease and then increase in secondary mental health services ‘community contacts’. Birth requiring intervention appeared to rise over time, particularly for elective and emergency Caesarean (3% and 7% across epochs). Unassisted vaginal birth decreased (by 2%). PPH appeared to have increased (by 1.5%) and NICU admission fell (by 2%).

**Fig. 1:**
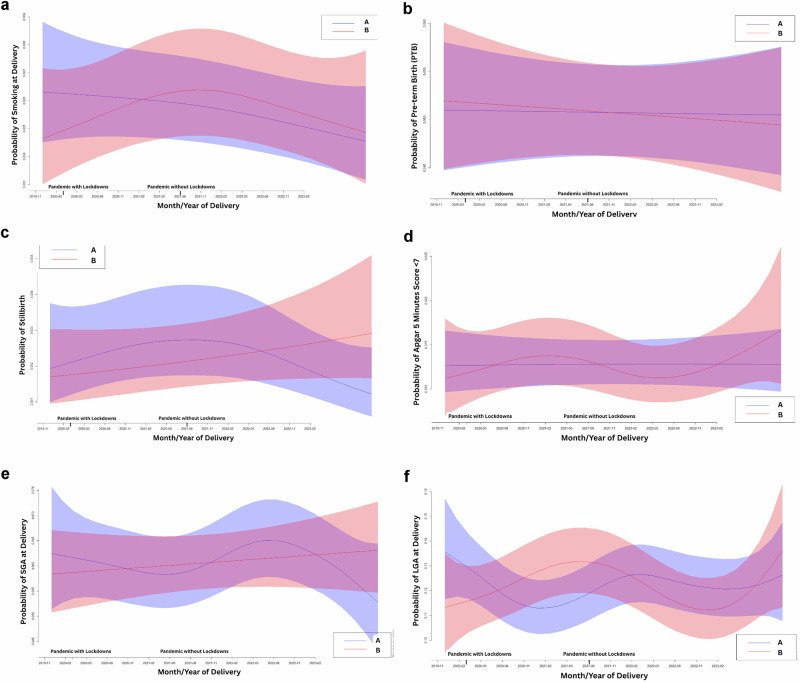
Probability of delivery outcomes with 95% confidence intervals, showing no change in trend over the pandemic period, for either site (all two-sided *p* values). **a** Smoking at delivery (p_A_ = 0.229and *p*_B_ = 0.322); **b** preterm birth (p_A_ = 0.837 and p_B_ = 0.662); **c** stillbirth (p_A_ = 0.124 and p_B_ = 0.234); **d** low 5-minute Apgar (p_A_ = 0.831 and p_B_ = 0.464); **e** Small-for-gestational age (SGA; *p*_A_ = 0.361 and p_B_ = 0.530); and **f** Large-for-gestational age (LGA; p_A_ = 0.268 and p_B_ = 0.198). Blue represents trends over time for site A while, red represent trends in site B. Generalised additive models (GAMs) were used to illustrate trends in outcomes over time, using flexible thin-plate regression splines stratified by site, from 1 May 2019 to 22 April 2023. The data were interpreted in terms of predicted means or probabilities of delivery outcomes, contingent upon the outcome distribution, per month/year of the study period. All outcomes are adjusted for variables based on published literature: ethnicity, Index of Multiple Deprivation quintile, gestational age at booking (weeks), smoking at booking, nulliparity, prior Caesarean, and month.

**Fig. 2:**
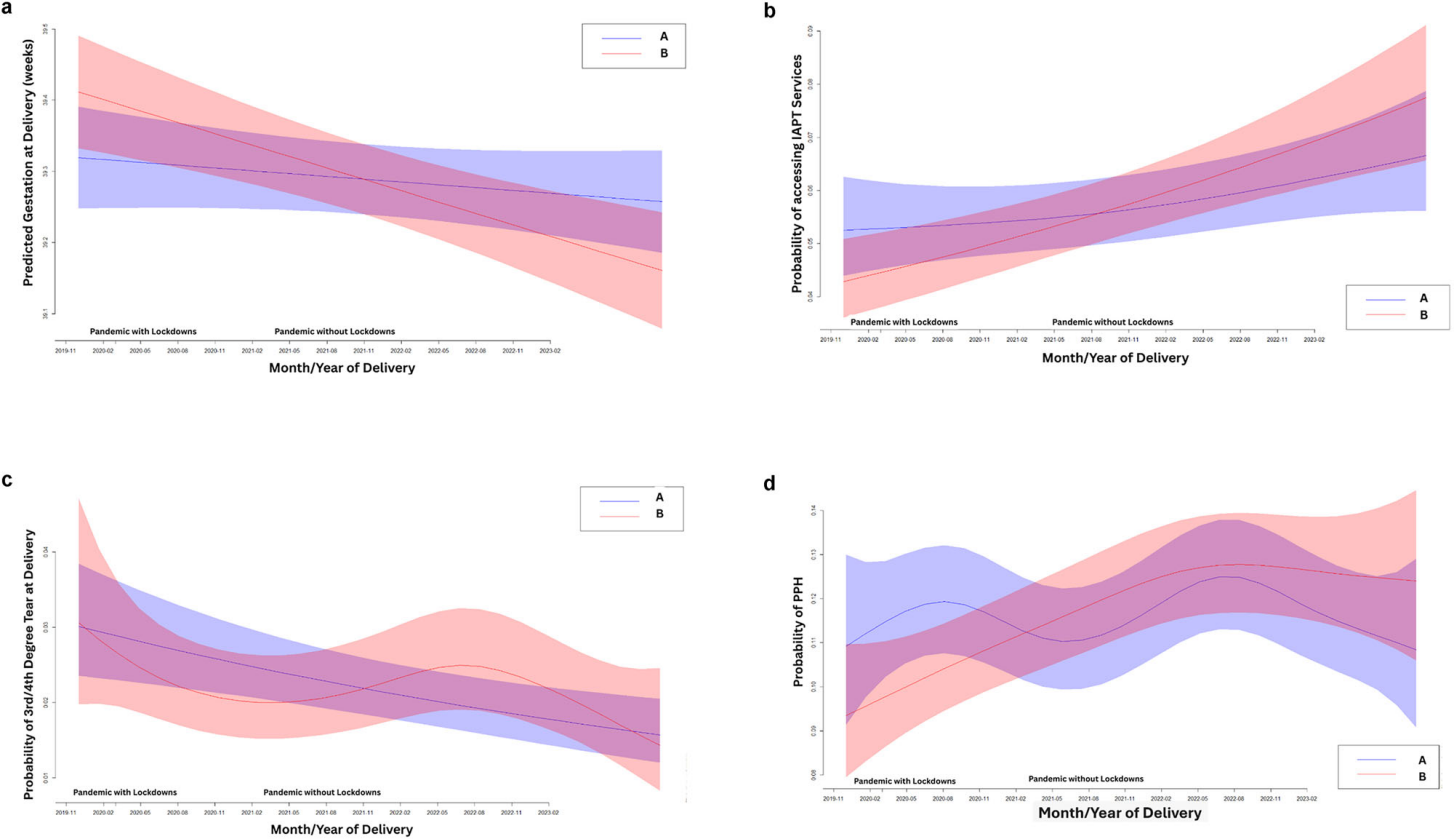
Probability of delivery outcomes with 95% confidence intervals, showing evidence of linear trend over the pandemic period at one site only (all two-sided *p* values). **a** Gestational age at birth (p_A_ = 0.228 and p_B_ < 0.001); **b** NHS ‘Talking Therapies’ (p_A_ =0.081 and p_B_ < 0.001); **c** vaginal tear (3rd or 4th degree) (p_A_ = 0.001 and p_B_ = 0.1883); and **d** postpartum haemorrhage (PPH; p_A_ = 0.387 and p_B_ = 0.003). Blue represents trends over time for site A while, red represent trends in site B. Generalised additive models (GAMs) were used to illustrate trends in outcomes over time, using flexible thin-plate regression splines stratified by site, from 1 May 2019 to 22 April 2023. The data were interpreted in terms of predicted means or probabilities of delivery outcomes, contingent upon the outcome distribution, per month/year of the study period. All outcomes are adjusted for variables based on published literature: ethnicity, Index of Multiple Deprivation quintile, gestational age at booking (weeks), smoking at booking, nulliparity, prior Caesarean, and month.

**Fig. 3:**
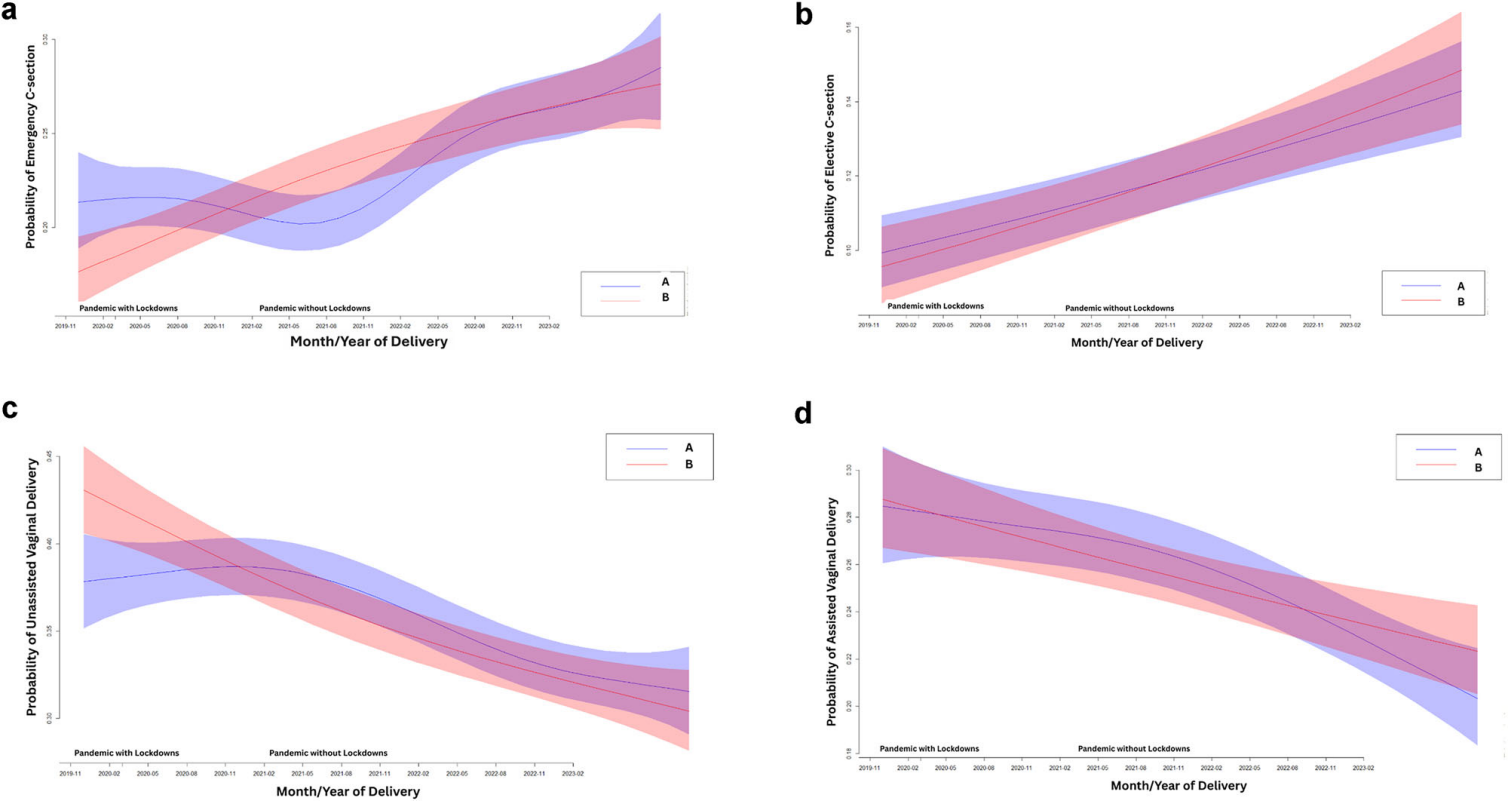
Probability of delivery outcomes with 95% confidence intervals, showing evidence of linear trend over the pandemic period at both sites (all two-sided *p* values). **a** Emergency Caesarean (p_A_ <0.001 and p_B_ < 0·001); **b** elective Caesarean (p_A_ <0.001 and p_B_ < 0.001); **c** unassisted vaginal birth (p_A_ <0.001 and p_B_ < 0.001); and **d** assisted vaginal birth specifically (p_A_ <0.001 and p_B_ < 0.001). Blue represents trends over time for site A while, red represent trends in site B. Generalised additive models (GAMs) were used to illustrate trends in outcomes over time, using flexible thin-plate regression splines stratified by site, from 1 May 2019 to 22 April 2023. The data were interpreted in terms of predicted means or probabilities of delivery outcomes, contingent upon the outcome distribution, per month/year of the study period. All outcomes are adjusted for variables based on published literature: ethnicity, Index of Multiple Deprivation quintile, gestational age at booking (weeks), smoking at booking, nulliparity, prior Caesarean, and month.

Table 2 presents adjusted outcome estimates from linear models (mean difference/OR, 95% CI and test for trend) across study epochs, and non-linear models by month (spline term p-values, sites A and B); unadjusted analyses are presented in Supplementary Table 2, which shows that unadjusted effects remained stable following adjustment for confounders across all outcomes, except with SGA. Figures 1–3 present visualisation of spline terms presented in Table 2, stratified by site and adjusted for confounders, as in analyses presented in Table 2 (where site was also an adjustment variable). Estimated degrees of freedom (EDF) are presented in Supplementary Table 3, and largely support our linear trends (as reflected by EDF values close to 1.0), and non-linear trends (as reflected by EDF values > 2.0), without any low *p*-values from the k-index test that may reflect a poorly-fitted model.

**Table 2:** Outcome event rates trends over time^a^

			Trend Test		Spline term^b^	
Outcomes	Pre-pandemic vs pandemic with lockdowns	Pandemic without lockdowns vs. pandemic with lockdowns	Linear *p*-value	Quadratic *p* value	Site A	Site B
Beta (95% CI)^c^						
Gestational age at birth (Wk)^c^	*−0.02 (−0.09, −0.04)*	*−0.07 (−0.13, −0.01)*	0.029	0.658	0.288	**<0.001**
aOR (95% CI)^d^	aORs (95% CI)^2^					
Smoker at birth^d^	0.88 (0.68−1.13)	0.81 (0.65, 1.01)	0.476	0.106	0.229	0.322
Mental Health Services						
Accessed NHS ‘Talking Therapies’^e^	0.97 (0.83−1.13)	*1.24 (1.09−1.40)*	**<0.001**	0.154	0.081	**<0.001**
Accessed ‘Community Contacts’^f^	*1.29 (1.09−1.53)*	*1.54 (1.33−1.78)*	0.018	**<0.001**	**<0.001**	0.022
Preterm birth	0.92 (0.81−1.03)	1.02 (0.92−1.14)	0.250	0.276	0.837	0.662
Labour induction	*1.27 (1.18−1.37)*	0.98 (0.92 – 1.04)	**<0.001**	**<0.001**	**<0.001**	0.092
Unassisted Vaginal Birth	*0.92 (0.85−0.98)*	*0.83 (0.78−0.88)*	**<0.001**	0.091	**<0.001**	**<0.001**
Interventional Delivery						
Emergency Caesarean	*1.08 (1.00−1.16)*	*1.25 (1.17−1.33)*	**<0.001**	0.016	**<0.001**	**<0.001**
Elective Caesarean	*1.12 (1.02−1.23)*	*1.20 (1.11−1.30)*	**<0.001**	0.377	**<0.001**	**<0.001**
Assisted Vaginal	0.95 (0.88−1.03)	*0.83 (0.77−0.89)*	**<0.001**	0.051	**<0.001**	**<0.001**
Vaginal tear (3^rd^/4^th^ degree)	*0.73 (0.57−0.93)*	1.03 (0.83−1.28)	**0.009**	0.087	**0.001**	0.188
PPH	0.99 (0.90−1.10)	*1.12 (1.02−1.22)*	0.031	0.154	0.387	**0.003**
Stillbirth	1.27 (0.83−2.00)	0.92 (0.64−1.33)	0.580	0.426	0.124	0.234
5-minute Apgar <7	1.02 (0.81−1.30)	1.00 (0.81−1.23)	0.843	0.908	0.831	0.464
SGA infants	0.93 (0.83−1.04)	1.08 (0.98−1.20)	0.966	0.121	0.361	0.530
LGA infants	1.02 (0.93−1.12)	0.98 (0.91−1.06)	0.993	0.579	0.268	0.198
Admitted to NICU	1.01 (0.90−1.13)	*0.70 (0.63−0.78)*	**<0.001**	**0.002**	**<0.00**1	0.377

CI (confidence interval), LGA (large-for-gestational age), NHS (National Health Service), NICU (neonatal intensive care unit), OR (odds ratio), PPH (postpartum haemorrhage), SGA (small-for-gestational age), Wk (weeks)

^a^95% confidence intervals that do not cross 1.0 are italics, and *p* values < 0.01 are bolded.

^b^Multivariable Generalised Additive Models’ *p*-value (two-sided), corresponding to site A and B spline terms for each outcome to evaluate the probability of stable trends across different sites. Models adjusted as below (c), except for site data, which were presented separately for sites A and B.

^c^Multivariable Linear Regression analysis for continuous outcomes presenting mean difference in estimates. All models were adjusted for the same minimal number of confounders: Data source (site A or B), Index of Multiple Deprivation Quintiles, Ethnicity, Gestation at booking (wks), Smoking at registration, Nulliparity, Month (for seasonality) and Previous Caesarean.

^d^Multivariable Logistic Regression for binary outcomes presenting OR with 95% CI. Models were adjusted as above^a^

^e^NHS ‘Talking Therapies’ are psychological therapies for anxiety and depression (https://www.england.nhs.uk/mental-health/adults/nhs-talking-therapies/).

^f^These are secondary mental health services community consultations.

Six outcomes showed stable probabilities over time: smoking at birth, preterm birth, stillbirth, 5-minute Apgar <7, and SGA and LGA infants, as illustrated by trend test results, and by spline terms for sites A and B (Table 2). Further graphical illustrations are provided in Fig. 1[a-f], which display flat (zero slope) trends with overlapping 95% CIs across both sites over the months/years, for each outcome. Spline terms for these outcomes indicated no evidence of linear or non-linear trends, at either site.

Linear trends were observed for several outcomes, with directions of overall effect shown by model estimates between epochs. Accessed ‘Talking Therapies’, emergency Caesareans, and elective Caesareans increased, while, unassisted vaginal birth, assisted vaginal birth, and 3rd or 4th-degree vaginal tears decreased (Table 2). Visualisation of spline terms by site revealed where linear trends were observed at only one site (but were stable at the other); gestational age at birth decreased only at site B (by approximately half a week, Fig. 2a), accessed ‘Talking Therapies’ increased only at site B (by ~4% to 9% probability, Fig. 2b), 3^rd^or 4^th^ degree vaginal tears decreased only at site A ( ~ 2% change in probability, Fig. 2c), and PPH increased only at site B (by ~4%, with a plateau during pandemic without lockdown, Fig. 2d); changes in gestational age at birth (Fig. 2a) and PPH (Fig. 2d) were very small, and were undetected by trend testing at a 1% significance level (Table 2); however, they were noted in real-time by site B, which attributed them to rising Caesarean rates, and instituted more consultant supervision of trainees during emergency Caesareans (see below), greater awareness of PPH risk factors, more frequent use of prophylactic uterotonics, and early escalation of PPH. Strong linear trends were observed at both sites for emergency and elective Caesareans (increasing y-axis probability limits from 20-35% [Fig. 3a] and 10-16% [Fig. 3b]), and for unassisted and assisted vaginal births (decreasing y-axis probability limits from 45 to 30% [Fig. 3c] and 30% to 18% [Fig. 3d]). However, notable GAM features include: (i) for Fig. 3a, some small fluctuations in emergency Caesareans at site A were undetected by the trend test analysis, but largely increased linearly; and (ii) for Fig. 3a, a pre-pandemic decrease in unassisted vaginal births at site B also began at site A during the first pandemic lockdown, with ongoing linear decreases thereafter.

**Fig. 4:**
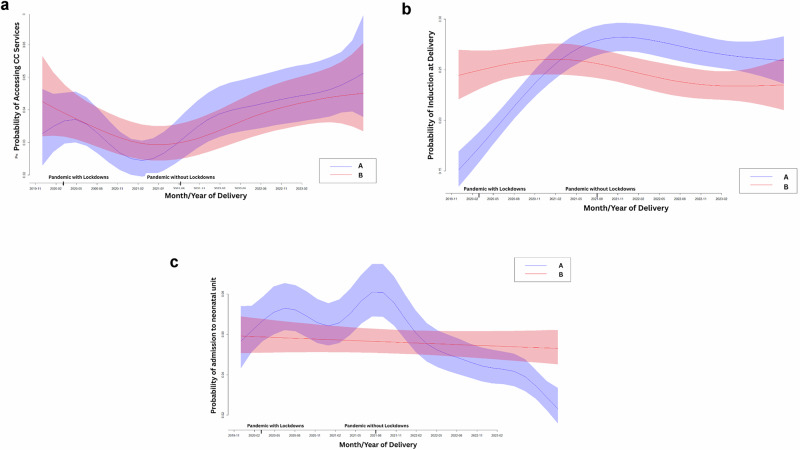
Probability of delivery outcomes with 95% confidence intervals, showing evidence of linear and non-linear trends over the pandemic periods but contrasting by site (all two-sided *p* values). **a** accessed secondary mental health services ‘Community contacts’ (p_A_ = <0.001 and p_B_ = 0.032); **b** labour induction (p_A_ <0.001 and p_B_ = 0.092); and **c** neonatal intensive care unit (NICU) admission (p_A_ = <0.001 and p_B_ = 0.3770). Blue represents trends over time for site A while, red represent trends in site B. Generalised additive models (GAMs) were used to illustrate trends in outcomes over time, using flexible thin-plate regression splines stratified by site, from 1 May 2019 to 22 April 2023. The data were interpreted in terms of predicted means or probabilities of delivery outcomes, contingent upon the outcome distribution, per month/year of the study period. All outcomes are adjusted for variables based on published literature: ethnicity, Index of Multiple Deprivation quintile, gestational age at booking (weeks), smoking at booking, nulliparity, prior Caesarean, and month.

Complex and non-linear trends were observed in secondary mental health services ‘community contacts’ (initially decreased by 2−3% during pandemic lockdowns [when reduced in availability], then increased), labour induction (sharp pre-pandemic increase from 15−28% probability and then plateaued at site B), and NICU admissions (some fluctuation at site A pre- and during pandemic lockdowns, then decreased from 8% mid-pandemic lockdowns to 2−3% probability). None of these patterns were consistent with seasonality. These trends were different at sites A and B (Table 2), except for accessed secondary mental health services ‘community contacts’, which, at both sites, decreased following the first pandemic lockdown, and then increased, surpassing pre-pandemic levels by the end of the pandemic (Fig. 4a).

### Sensitivity and further analyses

In the sensitivity analysis, 13/17 outcomes displayed no association between the odds of any outcome at birth, and the proportion of time spent in “pandemic with lockdowns”, across all three trimesters (Table 3). Four outcomes showed some such association: (i) access to ‘Community Contacts’ mental health services displayed a decreased odds associated with more time spent in ‘pandemic with lockdowns’ during the third trimester; (ii) labour induction displayed an increased odds with more time spent in lockdown in the third trimester; (iii) unassisted vaginal birth was less likely with more time spent in lockdown in the first trimester; and (iv) admission to the neonatal unit was more likely with higher proportions of first and third trimesters spent in lockdown, and less likely with high proportions of second trimester spent in lockdown. These results were consistent with the complex, site-specific changes reflected in the GAMs.

**Table 3:** Proportion of time spent in ‘pandemic with lockdowns’, by trimester*

Outcomes	Proportion within Trimester 1 (days)	Proportion with Trimester 2 (days)	Proportion within Trimester 3 (days)	
Beta (95% CI)^a^				
Gestational age at birth (Wk)^a^	−0.01 (−0.13, 0.10)	−0.01 (−0.17, 0.16)	0.01 (−0.10, 0.13)	
aORs (95% CI)^b^				
Smoker at birth^b^	1.04 (0.67, 1.611)	1.12 (0.58, 2.18)	1.18 (0.76, 1.82)	
Mental Health Services				
Accessed NHS ‘Talking Therapies’^c^	1.15 (0.89, 1.48)	0.93 (0.64, 1.37)	0.91 (0.70, 1.18)	
Accessed ‘Community Contacts’^d^	1.16 (0.88, 1.52)	1.00 (0.66, 1.51)	**0.67 (0.51, 0.88)**	
Preterm birth	1.03 (0.84, 1.27)	0.93 (0.68, 1.28)	1.03 (0.83, 1.27)	
Labour induction	1.07 (0.94, 1.22)	0.92 (0.76, 1.12)	**1.19 (1.05, 1.36)**	
Unassisted Vaginal Birth	**0.84 (0.74, 0.94)**	1.14 (0.95, 1.36)	1.05 (0.94, 1.19)	
Interventional Delivery				
Emergency Caesarean	1.12 (0.99, 1.27)	0.91 (0.75, 1.11)	0.91 (0.80, 1.03)	
Elective Caesarean	1.15 (0.98, 1.34)	0.94 (0.74, 1.20)	0.91 (0.78, 1.07)	
Assisted Vaginal	1.02 (0.88, 1.18)	0.96 (0.77, 1.21)	1.13 (0.97, 1.31)	
Vaginal tear (3^rd^/4^th^ degree)	1.17 (0.76, 1.78)	0.85 (0.45, 1.61)	0.86 (0.56, 1.31)	
PPH	0.99 (0.90 – 1.10)	1.12 (1.02, 1.22)	1.14 (0.96, 1.36)	
Stillbirth	1.27 (0.83 - 2.00)	0.92 (0.64, 1.33)	1.00 (0.77, 1.30)	
5-minute Apgar <7	1.02 (0.81 – 1.30)	1.00 (0.81, 1.23)	0.90 (0.76, 1.07)	
SGA infants	1.21 (0.99, 1.48)	0.85 (0.62, 1.15)	0.96 (0.78, 1.17)	
LGA infants	1.00 (0.85, 1.18)	1.08 (0.85, 1.37)	0.97 (0.83, 1.14)	
Admitted to NICU	**1.28 (1.04, 1.57)**	**0.65 (0.47, 0.89)**	**1.68 (1.36, 2.07)**	

CI (confidence interval), LGA (large-for-gestational age), NHS (National Health Service), NICU (neonatal intensive care unit), OR (odds ratio), PPH (postpartum haemorrhage), SGA (small-for-gestational age), Wk (weeks)

*95% confidence intervals that do not cross 1.0 and *p* values < 0.01 are bold.

^c^NHS ‘Talking Therapies’ are psychological therapies for anxiety and depression (https://www.england.nhs.uk/mental-health/adults/nhs-talking-therapies/),

^d^These are secondary mental health services community consultations.

^a^Multivariable Linear Regression analysis for continuous outcomes presenting mean difference in estimates. All models were adjusted for the same minimal number of confounders: Data source (site A or B), Index of Multiple Deprivation Quintiles, Ethnicity, Gestation at booking (wks), Smoking at registration, Nulliparity, Month (for seasonality) and Previous Caesarean.

^b^Multivariable Logistic Regression for binary outcomes presenting OR with 95% CI. Models were adjusted as above^a^.

Relationships between adjustment factors and outcomes are presented in Supplementary Table 4 and Supplementary Table 5. For all outcomes, significant associations were seen for minority ethnic groups (compared with White ethnicity) and nulliparity (vs. multiparity). For example, there was a 0.50−0.78 decreased odds of elective Caesarean across all ethnic minority groups (Black, Asian & Asian British, Mixed, and Multiple ethnic groups), compared with White ethnicity, and nulliparous (vs. multiparous) women showed a 4.58 increased odds of assisted vaginal birth. Other adjustment factors were associated with a number of adverse outcomes, such as a strong increased odds of elective Caesarean amongst those with the least (vs. most) deprived IMD, and for smokers (vs. non-smokers) at registration, a 0.41 decreased odds of LGA babies and 4.90 increased odds of accessing secondary mental health services ‘community contacts’. Eleven of 17 outcomes highlighted differences between the two sites (A vs. B). There was no evidence of seasonality in any maternal or birth outcomes.

For each outcome, interactions between time (pandemic epoch) and site, ethnicity, and IMD are presented in Supplementary Table 6**;** interactions with site were seen for induction, emergency Caesarean, PPH, and NICU admissions, as illustrated in Figs. 1–3. Similarly, interactions between time and ethnicity were seen for some outcomes, although most interactions were attributable to ‘other’ or ‘missing’ ethnicity categories (as seen in 13% of pregnancies pre-pandemic, 4.3% during pandemic lockdowns, and 6.8% during pandemic without lockdown). Only for labour induction during pandemic lockdowns was there an interaction with a specific ethnicity category (Black ethnicity). No interaction was seen between time and IMD for any outcome.

## Discussion

In more than 30,000 pregnancies from ethnically and socio-economically diverse South London, UK, we found that most pregnancy outcomes followed pre-pandemic temporal trends, either stable (e.g., preterm birth), increasing (e.g., elective Caesarean), or decreasing (e.g., assisted vaginal birth). While there was a clear pandemic-related decrease in accessing secondary mental health services ‘community contacts’ during the first pandemic lockdown, when those services were decreased in availability, other temporal trends (including labour induction and NICU admissions) were related to fluctuations at only one site, potentially due to local adaptations. We saw no evidence of seasonality, and any associations between outcomes and the proportion of time spent in a given trimester reflected the few observed complex site-specific changes illustrated by the GAMs.

Interaction tests between time and each site, ethnicity, and IMD quintiles supported the stratification of trends by data collection sites, highlighting their adaptations to pandemic impacts on maternal outcomes. While there was clear evidence of inequalities in outcomes for minority ethnic group (vs. White) women and those experiencing greater levels of deprivation, there was no compelling evidence that the pandemic exacerbated those inequalities.

In contrast to our study which covered the entire pandemic and examined monthly trends, the global impact of the pandemic on pregnancy outcomes has been examined primarily in the first pandemic year with lockdowns, or slightly beyond that into the summer of 2021, in studies in South London, North West England,^7^ England,^3^ or globally.^5^ While many studies found that the pandemic was associated with a decrease in preterm birth (overall and spontaneous),^4,5^ there was evidence of publication bias and heterogeneity.^19^ In a population-based study in England, pandemic lockdown (vs. pre-pandemic) was associated with small changes in outcomes, albeit small: fewer preterm births, more interventions (such as Caesarean or labour induction), and fewer SGA babies.^4^ Of note, if our approach had been to compare either pandemic lockdowns or the entire pandemic with pre-pandemic outcomes, we too would have described the pandemic to be associated with an increase in intervention (i.e., increase in emergency or elective Caesareans, and decrease in gestational age at birth and unassisted vaginal birth). Using interrupted time series analysis of data from North West England^7^ or Australia,^8^ outcomes were largely unchanged, including stillbirth, preterm birth, and abnormal fetal growth,^7^ or improvements in outcomes were reported, such as a reduction in labour induction for FGR which was not associated with a difference in FGR incidence or perinatal morbidity.^8^

As in our study, Gurol-Urganci et al. reported that some clinical outcomes (i.e., preterm births, Caesareans, and unassisted vaginal births) varied in the pandemic by ethnicity (White vs. minority ethnic groups), but not social deprivation (IMD quintiles 1−3 vs. 4−5).^4^ Our data add to this work, by clarifying that while minority group ethnicity and social deprivation are each associated with adverse outcomes, there is no specific ethnicity category for which pandemic-related effects were different. Rather, any interactions between ethnicity and the pandemic were, with one exception, due to unspecified or missing ethnicity, which was slightly more common pre-pandemic. Also, we have confirmed that there is no interaction between time and IMD, when quintiles 1−2 are compared with 3−5.

Our finding of ongoing increases in use of mental health treatment is consistent with published findings of a pandemic-related, negative legacy with regards to mental health. Pandemic-related social distancing restrictions had an adverse effect on women’s mental health before and after birth, related to restricted access to informal (family and friends) and formal (healthcare professional) support.^20^ Antenatally, women described feeling trapped, anxious, and abandoned during initial lockdown and its lifting,^21^ with prohibition of family from maternity wards particularly distressing whilst initial lockdown restrictions were being eased.^20,22^ A similar, cumulative, negative effect was seen on postpartum mental health,^6^ when pandemic social distancing restrictions could explain about one-third of the substantial increase in maternal depression (from 11% to 43%) and anxiety (from 18% to 61%), after accounting for current relevant diagnoses and other mental health risk factors.^23^ Younger women and sexual minority women were more likely to have postnatal anxiety, with younger participants reporting anxieties about infant safety and welfare, whilst lesbian, gay, bisexual, and pansexual participants struggled more with psychosocial adjustment to motherhood.^24^

Our finding that the vast majority of pregnancy outcomes followed pre-pandemic trends must be seen in light of the extensive, pandemic-related maternity service configurations in the UK. In line with recommendations from the Royal College of Obstetricians and Gynaecologists and Royal College of Midwives, a national survey documented that most UK maternity units reduced the number of antenatal contacts offered by any method, converted some antenatal appointments to remote consultations (particularly in the first and second trimesters, about which women reported mixed views,^2^ increased use of self-monitoring of blood pressure, modified screening for gestational diabetes, reduced the frequency of fetal growth surveillance by ultrasound, and reduced options for place of birth.^25^ Of note, there were few changes to labour induction indications or the offer of Caesarean by request.

When considering the impact of system-wide influences on pregnancy outcomes, our findings highlight the importance of evaluating longer-term temporal trends to contextualise short-term pregnancy outcome rates, as well as benchmarking between hospitals. Examining temporal trends in outcomes, and not just comparing outcome event rates between two time periods, minimises the risk of drawing spurious conclusions and overestimating the impact of acute events on outcomes, particularly as the relationships are complex between factors influencing maternity care and pregnancy outcomes, and can arise from individuals (e.g., care-seeking behaviour), care providers (e.g., staffing), or guidance, local or national. Benchmarking between sites, particularly temporally, may identify variations in outcomes and their underlying processes of care, with the goal of identifying best practice, optimising outcomes and minimising costs, as has been done successfully by others using detailed, routinely-collected data.^26^ All such activities are underpinned by a learning health system of data-driven learning, and rapid translation of learning into practice, to reduce the time to implementation; importantly, such an approach imbeds the engagement of key stakeholders, to co-design local solutions and influence ‘learning health system’ policy. By virtue of its principle of continuous learning, a learning health system prepares the health system for shocks, such as a the COVID-19 pandemic.

Strengths of our study include its large sample size and diversity of the study population in South London, UK, all based on routinely-collected data. We reported both physical and mental health outcomes. We covered the entire pandemic, facilitating our understanding of the impact of pandemic-related changes in care practices (and their withdrawal). Our multi-method analytic approach, which included multivariable regression, ordinal factor trend testing, and GAMs, allowed us to examine both linear and non-linear outcome patterns over time. The complementary results from each method led to comparable conclusions, further strengthening our findings. This methodological approach, not commonly employed to address trends in pregnancy outcomes, enabled us to make the distinction between ongoing trends and fluctuations due to pandemic-related changes. Additionally, our analyses were adjusted for site and individual-level characteristics, and we evaluated potential interactions between time and confounders. Also, we consulted clinicians from each hospital to deepen interpretation and verify trends.

With respect to limitations of this study, our decision to classify the timing of pregnancies according to pandemic epoch of birth means that findings may reflect predominantly late pregnancy and intrapartum care, rather than that earlier in pregnancy. Whilst multiple outcomes were explored separately, and analyses adjusted for the same set of minimal confounders, we believe our results reflect a coherent pattern of the main processes at play across study epochs. However, given the complex nature of these outcomes, further work would be warranted to assess outcome-specific adjustments. There was a very low rate of SARS-CoV2 positivity, this may reflect under-reporting (as seen in other routinely-collected data^21^), however a similar prevalence of 0.1% has been reported by other cohorts in pregnant women during the pandemic;^7^ due to the low prevalence, this precluded an analysis of the impact of SARS-CoV2 positivity on pregnancy outcomes. Finally, we analysed raw, uncoded clinical data in eLIXIR-BiSL; while some data missingness was evident and the validity of all such data cannot be confirmed, this is the information on which clinical care was based in the index pregnancy, and will be based in future pregnancies.

## Conclusions

In this large, diverse cohort of pregnancies from South London, UK, our findings reveal that, despite the significant health system shock of the pandemic and the decrement in experiences of maternity care, the direct impact on pregnancy outcomes was limited. Overall, outcomes during the pandemic largely reflected pre-pandemic trends and did not exacerbate inequalities, demonstrating the resilience of maternity services during this challenging period.

## Supplementary information

Supplementary Information

Description of Additional Supplementary files

Supplementary Data 1

Supplementary Data 2

Supplementary Data 3

Supllementary Data 4

## Data Availability

The data accessed by eLIXIR-BiSL remain within an NHS firewall and governance is provided by the eLIXIR Oversight Committee reporting to relevant information governance clinical leads. Subject to these conditions, data access is encouraged and those interested should contact the eLIXIR-BiSL Chief Investigator (Professor Lucilla Poston; lucilla.poston@kcl.ac.uk) or the HDRUK Innovation Gateway (https://healthdatagateway.org/en/search?type=datasets&query=elixir&page=1). The source data for Fig. 1 are in Supplementary Data 1. The source data for Fig. 2 are in Supplementary Data 2. The source data for Fig. 3 are in Supplementary Data 3. The source data for Fig. 4 are in Supplementary Data 4.

## Code availability

The custom code used for data analysis in this study is available upon reasonable request to the corresponding author. Access will be provided in accordance with institutional and ethical guidelines.
