# Supplementary material for: Temporal trends in pregnancy outcomes during a health system shock
Source: Commun Med (Lond). 2026 May 7;6:391. doi: 10.1038/s43856-026-01493-x (PMC13365597; doi:10.1038/s43856-026-01493-x)

SUPPLEMENTARY INFORMATION

Document type	Title	Page number
**Supplementary Tables**		
Supplementary Table 1	Patient characteristics for reduced and full data (following removal of those with more than one pregnancy per woman within the study period)	2
Supplementary Table 2	Baseline characteristic and pregnancy outcome significance testing (unadjusted and adjusted analyses)	3
Supplementary Table 3	Estimated degrees of freedom for models	4
Supplementary Table 4	Multivariable regression estimates from confounders for each delivery indicator	5
Supplementary Table 5	Multivariable regression estimates from confounders for each delivery indicator	6
Supplementary Table 6	Interaction between COVID period (pre, lockdown, without lockdown) with data source, IMD, and ethnicity	7
**Supplementary Figures**		
Supplementary Figure 1	Data flow diagram for the eLIXIR-BiSL data linkage	8
Supplementary Figure 2	Participant flow diagram	9
Supplementary Figure 3	Tails of uneven delivery periods and points of truncation	10

**Supplementary Table 1**: Patient characteristics for reduced and full data (following removal of those with more than one pregnancy per woman within the study period)		
	Reduced data	Full data
Antenatal Booking	N = 31,411[1]	N = 34,518[1]
Pandemic Period by Delivery Date		
Pre-Pandemic	7,706 (25%)	8,520 (25%)
Pandemic with Lockdowns	10,137 (32%)	11,033 (32%)
Pandemic without lockdowns	13,568 (43%)	14,965 (43%)
Data_Source_DS1		
Site A*	18,728 (60%)	20,579 (60%)
Site B*	12,683 (40%)	13,939 (40%)
Gestation At Booking Weeks	9.00 (8.00, 12.00)	9.00 (8.00, 12.00)
Ethnicity		
White	15,887 (51%)	17,421 (50%)
Black/African/Caribbean/Black British	6,309 (20%)	7,076 (20%)
Indian (Asian or Asian British)	765 (2.4%)	832 (2.4%)
Mixed/multiple ethnic groups	1,636 (5.2%)	1,792 (5.2%)
Other Asian/Asian British	1,930 (6.1%)	2,094 (6.1%)
Pakistani (Asian or Asian British)	327 (1.0%)	380 (1.1%)
Any Other ethnic group	2,186 (7.0%)	2,389 (6.9%)
(Missing)	2,371 (7.5%)	2,534 (7.3%)
Index of Multiple Deprivation Quintile		
1(most deprived)	6,050 (19%)	6,682 (19%)
2	12,914 (41%)	14,212 (41%)
3	7,790 (25%)	8,521 (25%)
4	2,906 (9.3%)	3,196 (9.3%)
5 (least deprived)	1,210 (3.9%)	1,319 (3.8%)
Missing	541 (1.7%)	588 (1.7%)
Parity	0.00 (0.00, 1.00)	0.00 (0.00, 1.00)
Nulliparous		
No	14,185 (45%)	16,272 (47%)
Yes	17,226 (55%)	18,246 (53%)
Smoker at Booking		
No	30,285 (96%)	33,282 (96%)
Yes	1,126 (3.6%)	1,236 (3.6%)
Previous Caesarean		
No	26,936 (86%)	29,419 (85%)
Yes	4,475 (14%)	5,099 (15%)
[1]n (%); Median (Q1, Q3)		

** Site A and Site B are maternity care hospitals contributing data to the eLIXIR-BiSL Partnership.*

**Supplementary Table 2**: Pregnancy outcome significance testing (unadjusted and adjusted analyses)*							
	Pre-pandemic (Epoch 1)	Pandemic + lock-downs (Epoch 2)	Pandemic without lockdowns(Epoch 3)	**Unadjusted** (OR, 95% confidence interval)		**Adjusted**[2,3] (OR, 95% confidence interval)	
Antenatal Booking	N = 7,706[1]	N = 10,137[1]	N = 13,568[1]	Epoch 1 vs. 2	Epoch 3 vs. 2	Epoch 1 vs. 2	Epoch 3 vs. 2
Pregnancy outcomes				Beta (95% CI)		Beta (95% CI)[1]	
Gestation At Birth (Wks)	39.00(38.00,40.00)	39.00(38.00,40.00)	39.00 (38.00,40.00)	0.00 (-0.06, 0.07)	-0.07 [-0.13, -0.02]	-0.02 (-0.09, -0.04)	-0.07 (-0.13, -0.01)
				**Odds ratio (95% CI)**		**Adjusted odds ratio (95% CI)** [2]	
Smoker At Birth	216 (2.8%)	278 (2.7%)	331 (2.4%)	0.98 (0.82, 1.17)	0.89 (0.75, 1.04)	0.88 (0.68 - 1.13)	0.81 (0.65, 1.01)
NHS 'Talking Therapies'†	297 (3.9%)	420 (4.1%)	655 (4.8%)	1.08 (0.93, 1.26)	1.17 (1.04, 1.33)	0.97 (0.83 - 1.13)	1.24 (1.09 – 1.40)
'Community contacts'‡	285 (3.7%)	298 (2.9%)	593 (4.4%)	0.79 (0.67, 0.93)	1.52 (1.32, 1.75)	1.29 (1.09, 1.53)	1.54 (1.33, 1.78)
Preterm birth	535 (6.9%)	634 (6.3%)	905 (6.7%)	0.89 (0.79, 1.01)	1.07 (0.96, 1.19)	0.92 (0.81, 1.03)	1.02 (0.92, 1.14)
Induction of labour	1,430 (19%)	2,323 (23%)	3,092 (23%)	1.30 (1.21, 1.40)	0.99 (0.93, 1.06)	1.27 (1.18, 1.37)	0.98 (0.92, 1.04)
Unassisted Vaginal Birth	3,862 (50%)	4,772 (47%)	5,734 (42%)	0.89 (0.84, 0.94)	0.82 (0.78, 0.87)	0.92 (0.85, 0.98)	0.83 (0.78, 0.88)
(Missing)	8 (0.1%)	28 (0.3%)	48 (0.4%)	-	-	-	-
Emergency Caesarean	1,473 (19%)	2,123 (21%)	3,466 (26%)	1.12 (1.04, 1.21)	1.30 (1.22, 1.38)	1.08 (1.00, 1.16)	1.25 (1.17, 1.33)
(Missing)	8 (0.1%)	28 (0.3%)	48 (0.4%)				
Elective Caesarean	1,115 (14%)	1,580 (16%)	2,369 (17%)	1.09 (1.01, 1.19)	1.15 (1.07, 1.23)	1.12 (1.02, 1.23)	1.20 (1.11, 1.30)
(Missing)	8 (0.1%)	28 (0.3%)	48 (0.4%)				
Assisted Vaginal birth	1,248 (16%)	1,634 (16%)	1,951 (14%)	1.00 (0.92, 1.08)	0.87 (0.81, 0.94)	0.95 (0.88, 1.03)	0.83 (0.77, 0.89)
(Missing)	8 (0.1%)	28 (0.3%)	48 (0.4%)				
Vaginal tear (3rd / 4th)	146 (1.9%)	144 (1.4%)	210 (1.5%)	0.75 (0.59, 0.94)	1.09 (0.88, 1.35)	0.73 (0.57, 0.93)	1.03 (0.83, 1.28)
Postpartum haemorrhage	731 (9.5%)	976 (9.6%)	1,479 (11%)	1.02 (0.92, 1.12)	1.15 (1.05, 1.25)	0.99 (0.90, 1.10)	1.12 (1.02, 1.22)
(Missing)	0 (0%)	0 (0%)	1 (<0.1%)				
Stillbirth	35 (0.5%)	55 (0.5%)	73 (0.5%)	1.20 (0.79, 1.84)	0.99 (0.70, 1.41)	1.27 (0.83, 2.00)	0.92 (0.64, 1.33)
5-min Apgar <7	121 (1.6%)	157 (1.5%)	224 (1.7%)	0.99 (0.78, 1.26)	1.07 (0.87, 1.32)	1.02 (0.81, 1.30)	1.00 (0.81, 1.23)
(Missing)	202 (2.6%)	298 (2.9%)	434 (3.2%)				
Small-for-gestational age	546 (7.1%)	681 (6.7%)	1,015 (7.5%)	0.94 (0.84, 1.06)	1.12 (1.02, 1.24)	0.93 (0.83, 1.04)	1.08 (0.98, 1.20)
(Missing)	19 (0.2%)	16 (0.2%)	40 (0.3%)				
Large-for-gestational age	959 (12%)	1,270 (13%)	1,612 (12%)	1.01 (0.92, 1.10)	0.94 (0.87, 1.02)	1.02 (0.93, 1.12)	0.98 (0.91, 1.06)
(Missing)	19 (0.2%)	16 (0.2%)	40 (0.3%)				
Admitted to neonatal ICU	576 (7.5%)	737 (7.3%)	759 (5.6%)	0.97 (0.87, 1.09)	0.76 (0.68, 0.84)	1.01 (0.90, 1.13)	0.70 (0.63, 0.78)

ICU (intensive care unit), NHS (National Health Service). *Highlighting=unadjusted (yellow) or adjusted (grey) analyses for which 95% CI of odds ratio did not cross 1.0. †Psychological therapies for anxiety and depression. ‡Secondary mental health community consultations. [1]n (%); Median (IQR) [2]Multivariable linear regression for continuous outcomes presenting mean difference in estimates. All models adjusted for: Data source (site A or B), Index of Multiple Deprivation, Ethnicity, Gestation at booking, Smoking, Nulliparity, Prior Caesarean. [3]Multivariable logistic regression for binary outcomes presenting odds ratio with 95% CI. Models were adjusted as above[2].

Supplementary Table 3: Estimated degrees of freedom for models*

	Estimated degrees of freedom (p value)		P values from Trend Test, Table 2, for reference		GAM p-values (spline terms) from Table 2, for reference)[1]	
	Data Source A	Data Source B	Linear	Quadratic	Site A	Site B
Gestation at Delivery	1 (p=0.41)	1 (p=0.48)	0.029	0.658	0.288	<0.001
Smoker at birth	1.38 (p=0.34)	1.88 (p=0.38)	0.476	0.106	0.229	0.322
Accessed NHS Talking Therapy	1.39 (p=0.94	1.01 (p=0.94)	<0.001	0.154	0.081	<0.001
Accessed community contacts	5.95 (p=0.53)	3.12 (p=0.66)	0.018	<0.001	<0.001	0.022
PTB	1.00 (p=0.41)	1.01 (p=0.43)	0.250	0.276	0.837	0.662
induced	2.87 (p=0.81)	2.54 (p=0.82)	<0.001	<0.001	<0.001	0.092
Unassisted vaginal birth	3.05 (p=0.14)	1.7 (p=0.22)	<0.001	0.091	<0.001	<0.001
Em CS	4.48 (p=0.75)	1.88 (p=0.66)	<0.001	0.016	<0.001	<0.001
E CS	1.01 (p=0.61)	1.00 (p=0.61)	<0.001	0.377	<0.001	<0.001
Assisted vaginal birth	2.07 (p=0.37)	1.00 (p=0.47)	<0.001	0.051	<0.001	<0.001
$3^{rd}/4^{th}$ degree tear	1.01 (p=0.22)	3.15 (p=0.18)	0.009	0.087	0.001	0.188
PPH	4.46 (p=0.44)	2.25 (p=0.25)	0.031	0.154	0.387	0.003
Stillbirth	2.06 (p=0.43)	1.01 (p=0.51)	0.580	0.426	0.124	0.234
5-min Apgar <7	1.05 (p=0.11)	2.92 (p=0.08)	0.843	0.908	0.831	0.464
SGA	3.31 (p=0.13)	1.01 (p=0.12)	0.966	0.121	0.361	0.530
LGA	4.19 (p=0.84)	3.95 (p=0.83)	0.993	0.579	0.268	0.198
Admitted	7.28 (p=0.34)	1.00 (p=0.41)	<0.001	0.002	<0.001	0.377

* The estimated degrees of freedom (EDF) and p value (2-sided) indicate results that are similar to the p values from the Trend Test and Spline Test from Table 2, for reference. EDF values close to 1.0 reflect linear trends, and those >2.0 support non-linear trends. Low p-values for the EDF may reflect a poorly-fitted model.

[1]Multivariable Generalised Additive Models' p-value (2-sided), corresponding to site A and B spline terms for each outcome to evaluate the probability of stable trends across different sites. All models were adjusted for Index of Multiple Deprivation Quintiles, Ethnicity, Gestation at booking (wks), Smoking at registration, Nulliparity, and Previous Caesarean, except for site data, which were presented separately for sites A and B

Supplementary Table 4: Multivariable regression estimates from confounders for each delivery indicator (outcomes 1- 8, continued in Supplementary Table 5)								
	Gestation at Delivery (Weeks)*	Smoker at Delivery	Accessed NHS 'Talking Therapies'	Accessed secondary mental health services	Preterm birth	Labour induction	Unassisted Vaginal Birth	Vaginal tear (3rd/4th degree)
Data Source								
Site A	— (—)		— (—)	— (—)	— (—)	— (—)	— (—)	— (—)
Site B	0.09 (0.04, 0.14)	1 (0.82, 1.22)	1.32 (1.18, 1.48)	1.28 (1.14, 1.44)	1.06 (0.97, 1.17)	0.91 (0.86, 0.96)	0.98(0.93,1.03)	0.89 (0.73, 1.07)
GA at booking (Wk)	-0.02(-0.03,-0.02)	1.02 (1.01, 1.04)	0.94 (0.93, 0.95)	0.99 (0.99, 1.00)	1.03 (1.03, 1.04)	1 (0.99, 1.00)	1 (1.0, 1.00)	1 (0.98, 1.01)
Ethnicity								
White	— (—)	— (—)	— (—)	— (—)	— (—)	— (—)	— (—)	— (—)
Black†	-0.2 (-0.42,-0.15)	0.75 (0.58, 0.97)	0.95 (0.82, 1.10)	1.34 (1.15, 1.56)	1.3 (1.15, 1.46)	1.32 (1.23, 1.43)	1.27(1.18,1.36)	1.05 (0.79, 1.37)
Asian/Asian British	-0.36(-0.55,-0.16)	0.53 (0.32, 0.85)	0.71 (0.57, 0.88)	0.73 (0.56, 0.93)	1.2 (1.02, 1.40)	1.14 (1.04, 1.26)	0.96(0.88,1.05)	1.93 (1.49, 2.48)
Multiple ethnicitys	-0.23(-0.47, 0.01)	1.18 (0.83, 1.68)	1.19 (0.94, 1.48)	1.35 (1.06, 1.71)	1.03 (0.83, 1.26)	1.05 (0.92, 1.19)	1.25(1.11,1.40)	0.86 (0.52, 1.34)
Any other group	-0.26(-0.47,-0.05)	0.59 (0.37, 0.91)	0.87 (0.68, 1.09)	0.96 (0.74, 1.23)	0.95 (0.78, 1.15)	1.12 (1.01, 1.25)	1.08(0.97,1.19)	1.08 (0.73, 1.55)
(Missing)	-0.24(-0.45,-0.02)	0.79 (0.53, 1.15)	0.79 (0.61, 0.99)	0.74 (0.56, 0.95)	1.22 (1.03, 1.43)	0.88 (0.78, 0.99)	1.23(1.12,1.36)	1.52 (1.10, 2.05)
IMD Quintile								
1 (most deprived)	— (—)	— (—)	— (—)	— (—)	— (—)	— (—)	— (—)	— (—)
2	0.07 (-0.04, 0.17)	0.91 (0.72, 1.15)	1.06 (0.91, 1.23)	1.05 (0.90, 1.24)	0.91 (0.81, 1.03)	1 (0.92, 1.07)	1.02(0.95,1.09)	1.04 (0.81, 1.36)
3	0.11 (0.00, 0.22)	0.74 (0.55, 0.99)	1 (0.85, 1.18)	1.02 (0.85, 1.22)	0.85 (0.73, 0.97)	0.96 (0.88, 1.04)	0.86(0.80,0.93)	1.12 (0.85, 1.48)
4	-0.09(-0.22,0.04)	0.5 (0.31, 0.77)	0.66 (0.52, 0.85)	0.77 (0.59, 0.99)	1.03 (0.86, 1.23)	0.95 (0.85, 1.07)	0.86(0.78,0.95)	1 (0.69, 1.43)
5 (least deprived)	-0.39 -0.56,-0.21)	0.6 (0.28, 1.17)	0.69 (0.47, 0.98)	0.77 (0.51, 1.12)	1.34 (1.06, 1.68)	0.93 (0.79, 1.09)	0.74(0.64,0.86)	1.04 (0.61, 1.67)
(Missing)	-0.55(-0.89,-0.20)	1.11 (0.48, 2.34)	0.61 (0.32, 1.05)	0.71 (0.39, 1.19)	1.76 (1.34, 2.29)	0.91 (0.73, 1.13)	1.01(0.83,1.23)	0.8 (0.33, 1.62)
Nulliparous	0.23 (0.19, 0.28)	0.75 (0.61, 0.93)	1.14 (1.01, 1.30)	1.19 (1.04, 1.37)	1.19 (1.08, 1.33)	1.24 (1.16, 1.31)	0.16(0.15,0.17)	4.25 (3.24, 5.68)
Smoker at Booking	-0.7 (-0.83, -0.57)	224 (184, 274)	1.09 (0.81, 1.44)	4.9 (4.07, 5.86)	2.18 (1.81, 2.62)	1.21 (1.05, 1.40)	1.25(1.09,1.44)	0.73 (0.36, 1.29)
Previous Caesarean	`-0.43(-0.50,0.35)	0.76 (0.57, 1.01)	0.99 (0.82, 1.18)	1.03 (0.85, 1.25)	1.22 (1.06, 1.41)	0.22 (0.19, 0.25)	0.04(0.04,0.05)	0.72 (0.42, 1.19)
Month	0.1141	0.1561	0.7511	0.2518	0.2677	0.6061	0.7139	0.2974

GA (gestational age), IMD (index of multiple deprivation), NHS (National Health Service), Wk (weeks)

* Shading indicates 95% CI that do not cross 1.0, either below (in blue) or above (in yellow).

† Black ethnicity referred to Black (unspecified), Black African, Black Caribbean, or Black British

& Global p-value of covariate (from ANOVA overall F-test)

Supplementary Table 5: Multivariable regression fixed effect estimates from adjusters for each delivery indicator (outcomes 9 – 17)*									
	Emergency Caesarean	Elective Caesarean	Assisted Vaginal birth	PPH	Stillbirth	5-minute Apgar <7	SGA	LGA	Admitted to NICU
Data Source									
Site A	— (—)	— (—)	— (—)	— (—)	— (—)	— (—)	— (—)	— (—)	— (—)
Site B	0.95(0.90,1.00)	0.92(0.86,0.99)	1.13(1.11,1.27)	0.65(0.60,0.70)	1.72(1.24,2.38)	0.77(0.64,0.93)	1.0(0.91,1.09)	1.06(0.98,1.13)	0.61(0.55,0.68)
GA at booking (Wk)	1 (1.00, 1.01)	1.0(1.00,1.01)	0.99(0.99 1.00)	1 (0.99, 1.00)	1.05(1.03,1.07)	1.04(1.03,1.05)	1.01(1.00,1.02)	1.0(0.99,1.00)	1.05(1.04,1.05)
Ethnicity									
White	— (—)	— (—)	— (—)	— (—)	— (—)	— (—)	— (—)	— (—)	— (—)
Black†	1.74(1.62,1.87)	0.50(0.45,0.55)	0.51(0.45,0.56)	1.14(1.03,1.26)	1.4(0.89,2.17)	2.3(1.84,2.86)	2.18(1.95,2.45)	0.57(0.52,0.63)	1.01(0.78,1.31)
Asian/Asian British	1.31(1.19,1.43)	0.64(0.57,0.73)	1.06(0.96,1.18)	1.08(0.96,1.22)	1.05(0.52,1.95)	0.95(0.66,1.33)	2.22(1.94,2.55)	0.39(0.34,0.46)	1.02(0.69,1.49)
Mixed/multiple ethnic groups	1.20(1.06,1.36)	0.69(0.58,0.81)	0.76(0.65,0.88)	0.78(0.65,0.94)	1.2(0.49,2.49)	1.07(0.66,1.63)	1.53(1.26,1.86)	0.79(0.67,0.92)	1.88(1.27,2.73)
Any other	1.31(1.17,1.46)	0.67(0.58,0.77)	0.89(0.78,1.01)	0.93(0.79,1.08)	1.61(0.85,2.86)	0.99(0.65,1.45)	1.64(1.38,1.94)	0.7(0.60,0.80)	1.44(0.99,2.06)
(Missing)	0.99(0.88,1.11)	0.78(0.68,0.90)	0.92(0.82,1.04)	0.98(0.84,1.14)	3.27(2.08, 5.11)	1.46(1.04,2.02)	1.37(1.15,1.63)	0.75(0.65,0.86)	1.16(0.77,1.68)
IMD Quintile									
1 (most deprived	— (—)	— (—)	— (—)	— (—)	— (—)	— (—)	— (—)	— (—)	— (—)
2	0.92(0.85,0.99)	1.10(1.00,1.22)	1.01(0.92,1.11)	1.04(0.93,1.15)	0.83(0.52,1.35)	1.26(0.98,1.63)	0.94(0.84,1.05)	1.04(0.95,1.15)	1.08(0.88,1.32)
3	0.94(0.87,1.03)	1.28(1.15,1.42)	1.13(1.02,1.25)	1.02(0.91,1.15)	0.81(0.47,1.38)	1.17(0.88,1.57)	0.87(0.76,0.99)	1.03(0.92,1.14)	1.03(0.83,1.28)
4	0.84(0.75,0.93)	1.56(1.36,1.78)	1.07(0.94,1.21)	1.07(0.92,1.24)	0.88(0.41,1.73)	1.49(1.04,2.12)	0.83(0.69,0.99)	1.05(0.91,1.20)	1.08(0.83,1.40)
5 (least deprived)	0.94(0.80,1.10)	2.12(1.79,2.51)	0.75(0.62,0.91)	1.14(0.93, 1.38)	1.52(0.60, 3.35)	1.68(1.03,2.64)	0.73(0.55,0.95)	1.0(0.82,1.20)	1.23(0.89,1.68)
(Missing)	1.01(0.82,1.24)	1.05(0.78,1.39)	0.91(0.69,1.19)	1.24(0.94,1.62)	12.7(7.66,21.2)	1.41(0.74 2.47)	0.75(0.52,1.05)	1.04(0.77,1.37)	0.52(0.20,1.10)
Nulliparous	4.2 (3.89, 4.54)	1.92(1.75,2.12)	4.58(4.18,5.02)	1.76(1.60,1.93)	1.58(1.09,2.32)	1.51(1.22,1.88)	1.84(1.66,2.05)	0.61(0.56,0.66)	1.32(1.18,1.47)
Smoker at Booking	1.14(0.97,1.32)	0.71(0.58,0.87)	0.74(0.60,0.90)	0.73(0.57,0.91)	2.08(1.03,3.80	1.35(0.85,2.04)	2.79(2.33,3.31)	0.41(0.32,0.52)	1.61(1.30,1.96)
Previous Caesarean	3.28(2.98,3.61)	21.1(19.0,23.5)	0.81(0.69,0.96)	1.73(1.54,1.96)	1.28(0.74,2.17)	1.36(1.02,1.81)	1.01(0.86,1.18)	1.27(1.15,1.40)	1.39(1.21,1.61)
Month‡	0.0694	0.8537	0.4897	0.1190	0.9322	0.0313	0.2186	0.1756	0.2524

GA (gestational age), IMD (index of multiple deprivation), Wk (weeks)

** Shading indicates 95% CI that do not cross 1.0, either below (in blue) or above (in yellow).*

† Black ethnicity referred to Black (unspecified), Black African, Black Caribbean, or Black British

‡ Global p-value of covariate from ANOVA overall F-test.

Supplementary Table 6: Interactions between COVID period (pre, lockdown, without lockdown) and data source, IMD, and ethnicity (p-values)*

	Covariates		
Outcomes	Data Source	IMD Quintile	Ethnicity
Gestation At Delivery (Wks)	0.071	0.017	<0.001
Smoker At Birth	0.085	0.461	0.215
Mental Health Service accessed			
NHS 'Talking Therapies'*	0.069	0.132	0.767
'Community contacts'†	0.420	0.803	0.097
Preterm birth	0.428	0.530	0.006
Induction	<0.001	0.979	0.008
Unassisted Vaginal Birth	0.002	0.061	0.173
Interventional Delivery			
Emergency	<0.001	0.478	0.424
Elective	0.385	0.851	0.228
Assisted Vaginal	0.825	0.556	0.208
Vaginal tear (3rd / 4th)	0.373	0.429	0.080
PPH	0.002	0.447	0.477
Stillbirth	0.633	0.139	0.866
5-min Apgar <7	0.922	0.097	0.140
SGA	0.999	0.489	0.562
LGA	0.022	0.697	0.429
Admitted to NICU	0.006	0.866	<0.001

IMD (index of multiple deprivation), LGA (large-for-gestational age), NHS (National Health Service), NICU (neonatal intensive care unit), PPH (postpartum haemorrhage), SGA (small-for-gestational age), Wks (weeks)

* Using multivariable linear or logistic regression (for continuous or categorical variables, respectively) was used to test for each delivery outcome, for interactions between pandemic phase and: site, ethnicity, and IMD, to examine whether the pandemic exacerbated inequalities. P values (2-sided) <0.01 are shaded in yellow and were regarded as statistically significant.

† NHS 'Talking Therapies' are psychological therapies for anxiety and depression (https://www.england.nhs.uk/mental-health/adults/nhs-talking-therapies/)

‡ These are secondary mental health services community consultations.

Supplementary Figure 1: Data flow diagram for the eLIXIR-BiSL data linkage

LEGEND: Data flow for the eLIXIR-BiSL data linkage is described within the figure, according to seven steps, that beings with creation by the Clinical Data Linkage Service of identifiers, and ends with the linked dataset.

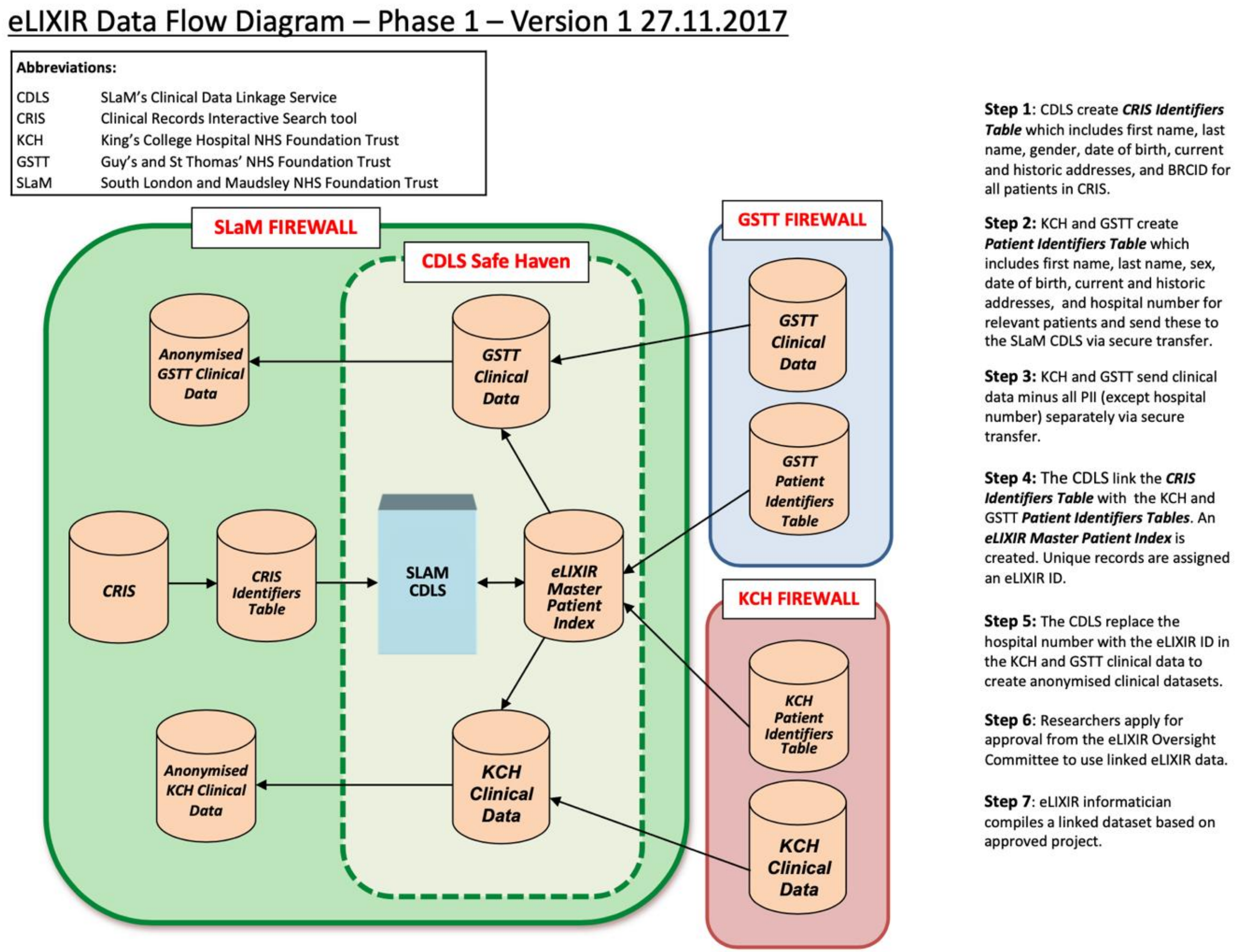

Supplementary Figure 2: Participant flow diagram and number of participants post-linkage

LEGEND: Linkage of maternity records from antenatal booking to birth.

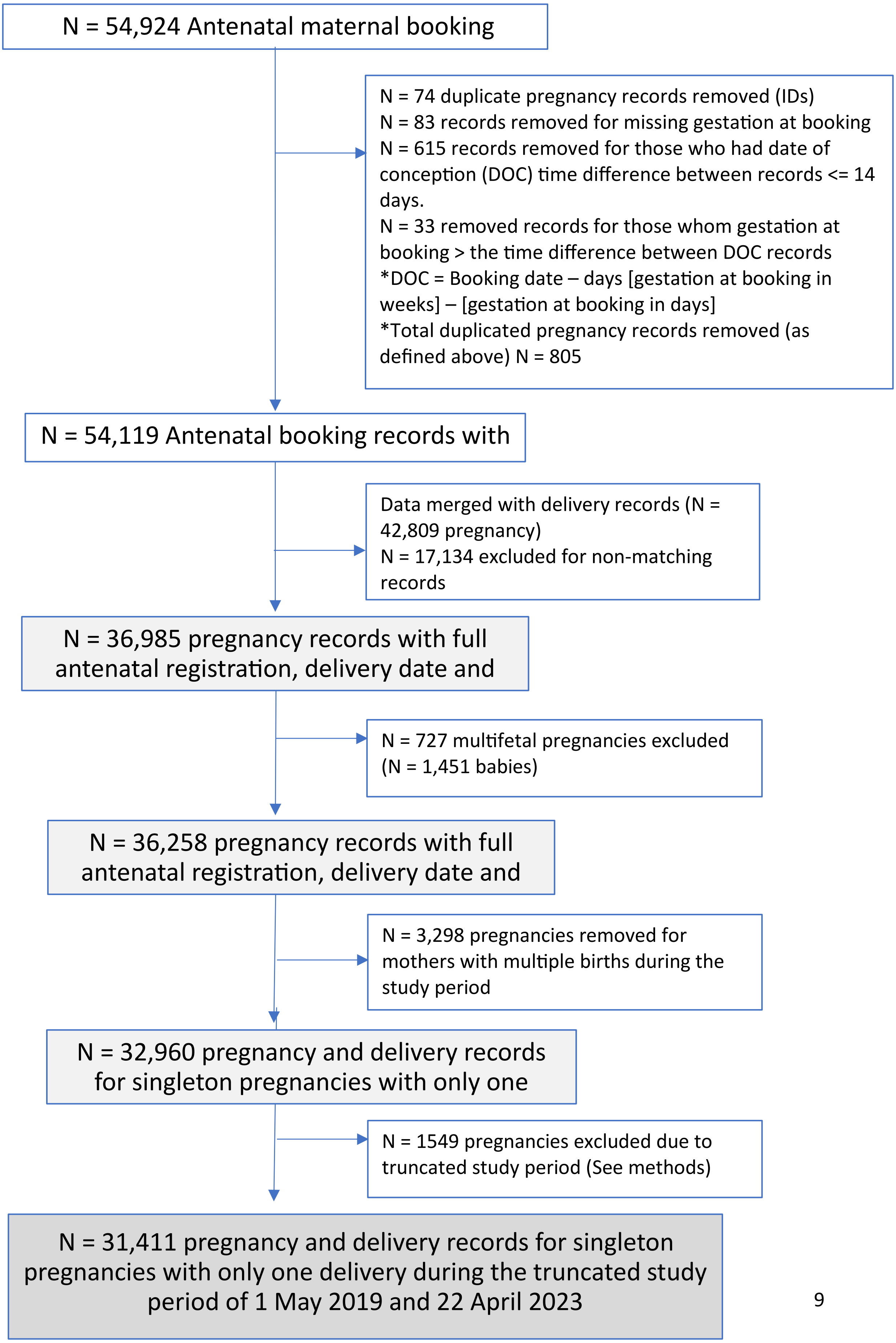

Supplementary Figure 3: Tails of uneven delivery periods and points of truncation.

LEGEND: Data truncation to restrict data to those with stable numbers of births.

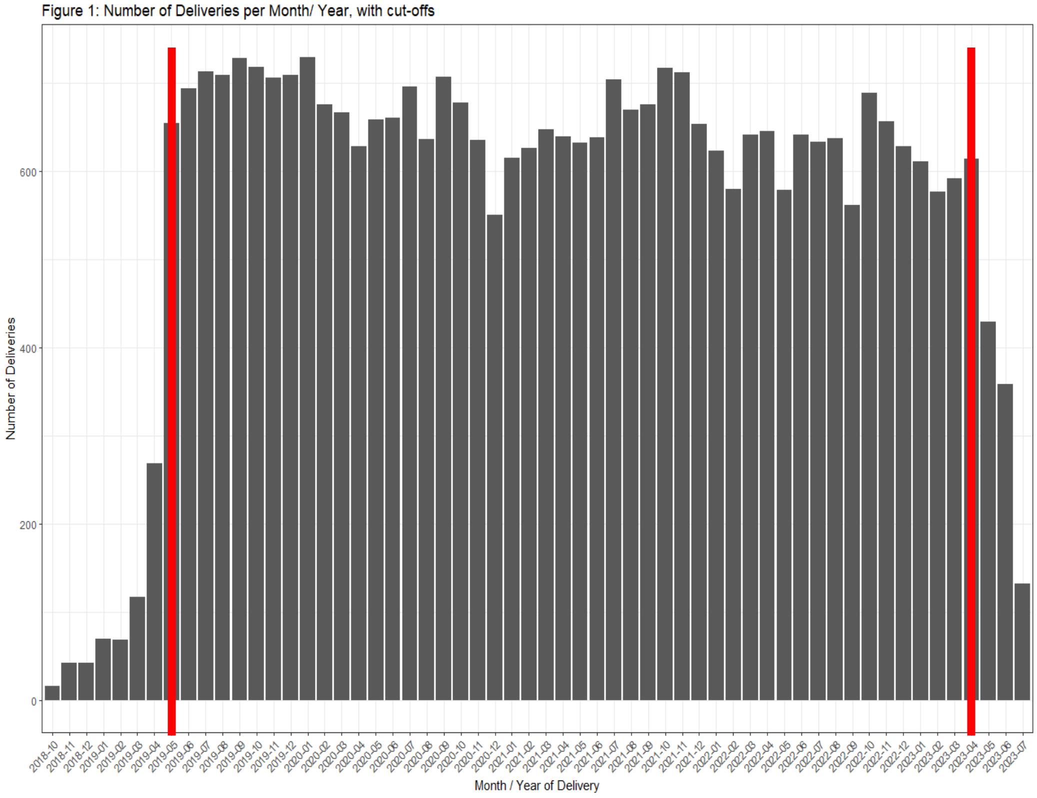

Supplement: Supplementary file 1 — Supplementary Information [file 43856_2026_1493_MOESM1_ESM.pdf]
