# Supplementary material for: Temporal trends in pregnancy outcomes during a health system shock
Source: Commun Med (Lond). 2026 May 7;6:391. doi: 10.1038/s43856-026-01493-x (PMC13365597; doi:10.1038/s43856-026-01493-x)
Supplement: Supplementary file 2 — Description of Additional Supplementary files [file 43856_2026_1493_MOESM2_ESM.docx]

**Description of Additional Supplementary Files**

File name: Supplementary Data 1-4

Description: Source data for Figure 1-4
