# Supplementary material for: Temporal trends in pregnancy outcomes during a health system shock
Source: Commun Med (Lond). 2026 May 7;6:391. doi: 10.1038/s43856-026-01493-x (PMC13365597; doi:10.1038/s43856-026-01493-x)
Supplement: Supplementary file 3 — Supplementary Data 1 [file 43856_2026_1493_MOESM3_ESM.docx]

**SUPPLEMENTARY DATA 1**

Title: Source data for Figure 1

Legend: N/month outcomes for each of the following outcomes, for the total study population and for sites A and B separately: (a) smoking at delivery; (b) preterm birth; (c) stillbirth; (d) low 5-minute Apgar; (e) Small-for-gestational age; and (f) Large-for-gestational age. The N for outcomes with fewer than 10 events has been suppressed.

Supplementary Data 1(a): **smoking at delivery**

| Month/Year | Site A | | Site B | |
| --- | --- | --- | --- | --- |
|  | n smoking at birth | N total | n smoking at birth | N total |
| 2019-11 | 14 | 305 | <10 | 401 |
| 2019-12 | <10 | 313 | 13 | 396 |
| 2020-01 | <10 | 290 | 16 | 440 |
| 2020-02 | <10 | 274 | 17 | 402 |
| 2020-03 | <10 | 293 | <10 | 374 |
| 2020-04 | <10 | 255 | <10 | 374 |
| 2020-05 | <10 | 301 | <10 | 358 |
| 2020-06 | <10 | 270 | 11 | 391 |
| 2020-07 | 10 | 292 | <10 | 404 |
| 2020-08 | 11 | 247 | 10 | 390 |
| 2020-09 | 10 | 288 | 13 | 419 |
| 2020-10 | 10 | 289 | 14 | 389 |
| 2020-11 | <10 | 260 | <10 | 376 |
| 2020-12 | <10 | 213 | <10 | 338 |
| 2021-01 | 11 | 268 | <10 | 347 |
| 2021-02 | <10 | 266 | 15 | 361 |
| 2021-03 | <10 | 279 | 16 | 369 |
| 2021-04 | <10 | 247 | <10 | 393 |
| 2021-05 | 11 | 256 | <10 | 377 |
| 2021-06 | <10 | 266 | 10 | 373 |
| 2021-07 | <10 | 276 | <10 | 428 |
| 2021-08 | <10 | 268 | 10 | 402 |
| 2021-09 | <10 | 299 | 13 | 377 |
| 2021-10 | <10 | 292 | 11 | 425 |
| 2021-11 | <10 | 291 | <10 | 421 |
| 2021-12 | <10 | 261 | 10 | 393 |
| 2022-01 | <10 | 234 | <10 | 390 |
| 2022-02 | 12 | 218 | <10 | 362 |
| 2022-03 | <10 | 229 | 11 | 413 |
| 2022-04 | <10 | 260 | <10 | 386 |
| 2022-05 | <10 | 241 | 12 | 338 |
| 2022-06 | <10 | 242 | 11 | 400 |
| 2022-07 | <10 | 251 | <10 | 383 |
| 2022-08 | <10 | 222 | <10 | 416 |
| 2022-09 | 11 | 211 | 12 | 351 |
| 2022-10 | <10 | 259 | 13 | 430 |
| 2022-11 | <10 | 241 | 10 | 416 |
| 2022-12 | <10 | 244 | 11 | 385 |
| 2023-01 | <10 | 241 | <10 | 370 |
| 2023-02 | <10 | 230 | <10 | 347 |
| 2023-03 | <10 | 221 | <10 | 371 |
| 2023-04 | <10 | 220 | <10 | 394 |

Supplementary Data 1(b): **preterm birth**

| Month/year | Site A | | Site B | |
| --- | --- | --- | --- | --- |
|  | n preterm birth | Total | n preterm birth | Total |
| 2019-11 | 26 | 401 | 11 | 305 |
| 2019-12 | 39 | 396 | 25 | 313 |
| 2020-01 | 28 | 440 | 21 | 290 |
| 2020-02 | 33 | 402 | 26 | 274 |
| 2020-03 | 31 | 374 | 25 | 293 |
| 2020-04 | 22 | 374 | 16 | 255 |
| 2020-05 | 22 | 358 | 20 | 301 |
| 2020-06 | 23 | 391 | 16 | 270 |
| 2020-07 | 28 | 404 | 21 | 292 |
| 2020-08 | 17 | 390 | 16 | 247 |
| 2020-09 | 15 | 419 | 15 | 288 |
| 2020-10 | 26 | 389 | 15 | 289 |
| 2020-11 | 22 | 376 | 14 | 260 |
| 2020-12 | 21 | 338 | 16 | 213 |
| 2021-01 | 26 | 347 | 29 | 268 |
| 2021-02 | 23 | 361 | 13 | 266 |
| 2021-03 | 18 | 369 | 18 | 279 |
| 2021-04 | 26 | 393 | 17 | 247 |
| 2021-05 | 22 | 377 | 19 | 256 |
| 2021-06 | 27 | 373 | 18 | 266 |
| 2021-07 | 30 | 428 | 19 | 276 |
| 2021-08 | 40 | 402 | 22 | 268 |
| 2021-09 | 24 | 377 | 18 | 300 |
| 2021-10 | 23 | 425 | 16 | 292 |
| 2021-11 | 29 | 421 | 12 | 291 |
| 2021-12 | 20 | 393 | 17 | 261 |
| 2022-01 | 20 | 390 | 16 | 234 |
| 2022-02 | 28 | 362 | 13 | 218 |
| 2022-03 | 23 | 413 | 19 | 229 |
| 2022-04 | 23 | 386 | 18 | 260 |
| 2022-05 | 24 | 338 | 13 | 241 |
| 2022-06 | 22 | 400 | 17 | 242 |
| 2022-07 | 15 | 383 | 20 | 251 |
| 2022-08 | 40 | 416 | 16 | 222 |
| 2022-09 | 28 | 351 | 13 | 211 |
| 2022-10 | 36 | 430 | 17 | 259 |
| 2022-11 | 28 | 416 | 19 | 241 |
| 2022-12 | 17 | 385 | 15 | 244 |
| 2023-01 | 30 | 370 | 13 | 241 |
| 2023-02 | 26 | 347 | 16 | 230 |
| 2023-03 | 24 | 371 | 10 | 221 |
| 2023-04 | 30 | 394 | 19 | 220 |

Supplementary Data 1(c): **stillbirth**

| Month/year | Site A | | Site B | |
| --- | --- | --- | --- | --- |
|  | n stillbirth | Total | n stillbirth | Total |
| 2019-11 | <10 | 401 | <10 | 305 |
| 2019-12 | <10 | 396 | <10 | 313 |
| 2020-01 | <10 | 440 | <10 | 290 |
| 2020-02 | <10 | 402 | <10 | 274 |
| 2020-03 | <10 | 374 | <10 | 293 |
| 2020-04 | <10 | 374 | <10 | 255 |
| 2020-05 | <10 | 358 | <10 | 301 |
| 2020-06 | <10 | 391 | <10 | 270 |
| 2020-07 | <10 | 404 | <10 | 292 |
| 2020-08 | <10 | 390 | <10 | 247 |
| 2020-09 | <10 | 419 | <10 | 288 |
| 2020-10 | <10 | 389 | <10 | 289 |
| 2020-11 | <10 | 376 | <10 | 260 |
| 2020-12 | <10 | 338 | <10 | 213 |
| 2021-01 | <10 | 347 | <10 | 268 |
| 2021-02 | <10 | 361 | <10 | 266 |
| 2021-03 | <10 | 369 | <10 | 279 |
| 2021-04 | <10 | 393 | <10 | 247 |
| 2021-05 | <10 | 377 | <10 | 256 |
| 2021-06 | <10 | 373 | <10 | 266 |
| 2021-07 | <10 | 428 | <10 | 276 |
| 2021-08 | <10 | 402 | <10 | 268 |
| 2021-09 | <10 | 377 | <10 | 299 |
| 2021-10 | <10 | 425 | <10 | 292 |
| 2021-11 | <10 | 421 | <10 | 291 |
| 2021-12 | <10 | 393 | <10 | 261 |
| 2022-01 | <10 | 390 | <10 | 234 |
| 2022-02 | <10 | 362 | <10 | 218 |
| 2022-03 | <10 | 413 | <10 | 229 |
| 2022-04 | <10 | 386 | <10 | 260 |
| 2022-05 | <10 | 338 | <10 | 241 |
| 2022-06 | <10 | 400 | <10 | 242 |
| 2022-07 | <10 | 383 | <10 | 251 |
| 2022-08 | <10 | 416 | <10 | 222 |
| 2022-09 | <10 | 351 | <10 | 211 |
| 2022-10 | <10 | 430 | <10 | 259 |
| 2022-11 | <10 | 416 | <10 | 241 |
| 2022-12 | <10 | 385 | <10 | 244 |
| 2023-01 | <10 | 370 | <10 | 241 |
| 2023-02 | <10 | 347 | <10 | 230 |
| 2023-03 | <10 | 371 | <10 | 221 |
| 2023-04 | <10 | 394 | <10 | 220 |

Supplementary Data 1(d): **Low 5-minute Apgar score**

| Month/year | Site A | | Site B | |
| --- | --- | --- | --- | --- |
|  | n low 5-min Apgar | Total | n low 5-min Apgar | Total |
| 2019-11 | <10 | 394 | <10 | 294 |
| 2019-12 | <10 | 388 | <10 | 300 |
| 2020-01 | <10 | 433 | <10 | 276 |
| 2020-02 | <10 | 394 | <10 | 266 |
| 2020-03 | <10 | 366 | <10 | 283 |
| 2020-04 | <10 | 367 | <10 | 246 |
| 2020-05 | <10 | 352 | <10 | 292 |
| 2020-06 | <10 | 386 | <10 | 255 |
| 2020-07 | <10 | 392 | <10 | 281 |
| 2020-08 | <10 | 382 | <10 | 233 |
| 2020-09 | <10 | 415 | <10 | 275 |
| 2020-10 | <10 | 381 | <10 | 280 |
| 2020-11 | <10 | 367 | <10 | 248 |
| 2020-12 | <10 | 333 | <10 | 204 |
| 2021-01 | <10 | 338 | <10 | 256 |
| 2021-02 | <10 | 354 | <10 | 256 |
| 2021-03 | 14 | 363 | <10 | 272 |
| 2021-04 | <10 | 385 | <10 | 235 |
| 2021-05 | <10 | 368 | <10 | 239 |
| 2021-06 | <10 | 364 | <10 | 254 |
| 2021-07 | <10 | 417 | <10 | 261 |
| 2021-08 | 12 | 390 | <10 | 252 |
| 2021-09 | <10 | 367 | <10 | 289 |
| 2021-10 | <10 | 413 | <10 | 279 |
| 2021-11 | <10 | 407 | <10 | 284 |
| 2021-12 | <10 | 382 | <10 | 249 |
| 2022-01 | <10 | 380 | <10 | 227 |
| 2022-02 | <10 | 350 | <10 | 210 |
| 2022-03 | 11 | 405 | <10 | 212 |
| 2022-04 | <10 | 381 | <10 | 244 |
| 2022-05 | <10 | 328 | <10 | 231 |
| 2022-06 | 10 | 395 | <10 | 230 |
| 2022-07 | <10 | 370 | <10 | 239 |
| 2022-08 | 14 | 403 | <10 | 214 |
| 2022-09 | <10 | 346 | <10 | 206 |
| 2022-10 | <10 | 415 | <10 | 251 |
| 2022-11 | <10 | 403 | <10 | 232 |
| 2022-12 | <10 | 369 | <10 | 233 |
| 2023-01 | <10 | 362 | <10 | 236 |
| 2023-02 | <10 | 341 | <10 | 221 |
| 2023-03 | <10 | 363 | <10 | 215 |
| 2023-04 | <10 | 387 | <10 | 211 |

Supplementary Data 1(e): **Small-for-gestational age (SGA) infants**

| Month/year | Site A | | Site B | |
| --- | --- | --- | --- | --- |
|  | SGA infants | Total | SGA infants | Total |
| 2019-11 | 28 | 399 | 24 | 304 |
| 2019-12 | 32 | 395 | 23 | 312 |
| 2020-01 | 35 | 440 | 23 | 289 |
| 2020-02 | 32 | 400 | 19 | 272 |
| 2020-03 | 22 | 374 | 27 | 292 |
| 2020-04 | 24 | 373 | 17 | 255 |
| 2020-05 | 30 | 357 | 19 | 300 |
| 2020-06 | 22 | 390 | 14 | 270 |
| 2020-07 | 33 | 404 | 17 | 292 |
| 2020-08 | 21 | 390 | 12 | 247 |
| 2020-09 | 39 | 417 | 19 | 288 |
| 2020-10 | 21 | 388 | 18 | 288 |
| 2020-11 | 21 | 375 | 17 | 260 |
| 2020-12 | 22 | 338 | 18 | 213 |
| 2021-01 | 22 | 346 | 15 | 268 |
| 2021-02 | 22 | 359 | 18 | 265 |
| 2021-03 | 27 | 369 | 19 | 279 |
| 2021-04 | 31 | 393 | 19 | 247 |
| 2021-05 | 23 | 376 | 15 | 256 |
| 2021-06 | 24 | 373 | 17 | 264 |
| 2021-07 | 38 | 428 | 25 | 275 |
| 2021-08 | 24 | 401 | 15 | 267 |
| 2021-09 | 35 | 376 | 23 | 298 |
| 2021-10 | 40 | 423 | 13 | 291 |
| 2021-11 | 32 | 419 | 18 | 290 |
| 2021-12 | 27 | 393 | 23 | 260 |
| 2022-01 | 40 | 390 | 21 | 233 |
| 2022-02 | 25 | 359 | 15 | 217 |
| 2022-03 | 37 | 412 | 23 | 229 |
| 2022-04 | 27 | 385 | 20 | 260 |
| 2022-05 | 20 | 337 | 10 | 239 |
| 2022-06 | 33 | 397 | 21 | 240 |
| 2022-07 | 18 | 383 | 22 | 250 |
| 2022-08 | 34 | 414 | 23 | 222 |
| 2022-09 | 35 | 351 | 20 | 211 |
| 2022-10 | 37 | 429 | 17 | 259 |
| 2022-11 | 26 | 415 | 19 | 241 |
| 2022-12 | 26 | 383 | 18 | 244 |
| 2023-01 | 27 | 369 | 25 | 241 |
| 2023-02 | 29 | 347 | 13 | 230 |
| 2023-03 | 23 | 371 | 15 | 220 |
| 2023-04 | 20 | 393 | <10 | 217 |

Supplementary Data 1(f): **large-for-gestational age (LGA) infants**

| Month/year | Site A | | Site B | |
| --- | --- | --- | --- | --- |
|  | n LGA infants | Total | n LGA infants | Total |
| 2019-11 | 54 | 399 | 25 | 304 |
| 2019-12 | 52 | 395 | 37 | 312 |
| 2020-01 | 52 | 440 | 40 | 289 |
| 2020-02 | 45 | 400 | 36 | 272 |
| 2020-03 | 35 | 374 | 32 | 292 |
| 2020-04 | 41 | 373 | 32 | 255 |
| 2020-05 | 37 | 357 | 50 | 300 |
| 2020-06 | 39 | 390 | 36 | 270 |
| 2020-07 | 49 | 404 | 47 | 292 |
| 2020-08 | 35 | 390 | 25 | 247 |
| 2020-09 | 56 | 417 | 38 | 288 |
| 2020-10 | 41 | 388 | 49 | 288 |
| 2020-11 | 42 | 375 | 38 | 260 |
| 2020-12 | 33 | 338 | 24 | 213 |
| 2021-01 | 40 | 346 | 29 | 268 |
| 2021-02 | 45 | 359 | 41 | 265 |
| 2021-03 | 40 | 369 | 49 | 279 |
| 2021-04 | 43 | 393 | 31 | 247 |
| 2021-05 | 53 | 376 | 37 | 256 |
| 2021-06 | 55 | 373 | 32 | 264 |
| 2021-07 | 49 | 428 | 40 | 275 |
| 2021-08 | 58 | 401 | 36 | 267 |
| 2021-09 | 54 | 376 | 31 | 298 |
| 2021-10 | 51 | 423 | 45 | 291 |
| 2021-11 | 46 | 419 | 39 | 290 |
| 2021-12 | 41 | 393 | 23 | 260 |
| 2022-01 | 42 | 390 | 27 | 233 |
| 2022-02 | 46 | 359 | 32 | 217 |
| 2022-03 | 51 | 412 | 17 | 229 |
| 2022-04 | 45 | 385 | 34 | 260 |
| 2022-05 | 39 | 337 | 29 | 239 |
| 2022-06 | 43 | 397 | 34 | 240 |
| 2022-07 | 47 | 383 | 29 | 250 |
| 2022-08 | 42 | 414 | 14 | 222 |
| 2022-09 | 42 | 351 | 24 | 211 |
| 2022-10 | 51 | 429 | 33 | 259 |
| 2022-11 | 45 | 415 | 26 | 241 |
| 2022-12 | 46 | 383 | 20 | 244 |
| 2023-01 | 36 | 369 | 32 | 241 |
| 2023-02 | 38 | 347 | 35 | 230 |
| 2023-03 | 41 | 371 | 32 | 220 |
| 2023-04 | 56 | 393 | 34 | 217 |
