# Supplementary material for: Temporal trends in pregnancy outcomes during a health system shock
Source: Commun Med (Lond). 2026 May 7;6:391. doi: 10.1038/s43856-026-01493-x (PMC13365597; doi:10.1038/s43856-026-01493-x)
Supplement: Supplementary file 4 — Supplementary Data 2 [file 43856_2026_1493_MOESM4_ESM.docx]

**SUPPLEMENTARY DATA 2**

Title: Source data for Figure 2

Legend: N/month outcomes for each of the following outcomes, for the total study population and for sites A and B separately: (a) gestational age at birth; (b) NHS ‘Talking Therapies’; (c) vaginal tear (3rd or 4th degree); and (d) postpartum haemorrhage. The N for outcomes with fewer than 10 events has been suppressed.

Supplementary Data 2(a): **gestational age at birth** (weeks)

| Month/ year | Site A | | | Site B | | |
| --- | --- | --- | --- | --- | --- | --- |
|  | Median  gestation | 25^th^ centile | 75^th^ centile | Median  gestation | 25^th^ centile | 75^th^ centile |
| 2019-11 | 401 | 39 | 38 | 39 | 39 | 40 |
| 2019-12 | 396 | 39 | 38 | 39 | 38 | 40 |
| 2020-01 | 440 | 39 | 38 | 39 | 38 | 40 |
| 2020-02 | 402 | 39 | 38 | 39 | 38 | 40 |
| 2020-03 | 374 | 39 | 38 | 39 | 38 | 40 |
| 2020-04 | 374 | 39 | 38 | 39 | 39 | 40 |
| 2020-05 | 358 | 39 | 38 | 39 | 39 | 40 |
| 2020-06 | 391 | 39 | 38 | 39 | 38 | 40 |
| 2020-07 | 404 | 39 | 38 | 40 | 39 | 40 |
| 2020-08 | 390 | 39 | 38 | 39 | 38 | 40 |
| 2020-09 | 419 | 39 | 38 | 39 | 38 | 40 |
| 2020-10 | 389 | 39 | 38 | 39 | 38 | 40 |
| 2020-11 | 376 | 39 | 38 | 39 | 38 | 40 |
| 2020-12 | 338 | 39 | 38 | 39 | 38 | 40 |
| 2021-01 | 347 | 39 | 38 | 39 | 38 | 40 |
| 2021-02 | 361 | 39 | 38 | 39 | 39 | 40 |
| 2021-03 | 369 | 39 | 38 | 39 | 38 | 40 |
| 2021-04 | 393 | 39 | 38 | 39 | 39 | 40 |
| 2021-05 | 377 | 39 | 38 | 39 | 38 | 40 |
| 2021-06 | 373 | 39 | 38 | 39 | 38 | 40 |
| 2021-07 | 428 | 39 | 38 | 39 | 38 | 40 |
| 2021-08 | 402 | 39 | 38 | 39 | 38 | 40 |
| 2021-09 | 377 | 39 | 38 | 39 | 39 | 40 |
| 2021-10 | 425 | 39 | 38 | 39 | 38 | 40 |
| 2021-11 | 421 | 39 | 38 | 39 | 39 | 40 |
| 2021-12 | 393 | 39 | 38 | 39 | 38 | 40 |
| 2022-01 | 390 | 39 | 38 | 39 | 38 | 40 |
| 2022-02 | 362 | 39 | 38 | 39 | 38 | 40 |
| 2022-03 | 413 | 39 | 38 | 39 | 38 | 40 |
| 2022-04 | 386 | 39 | 38 | 39 | 38 | 40 |
| 2022-05 | 338 | 39 | 38 | 39 | 38 | 40 |
| 2022-06 | 400 | 39 | 38 | 39 | 38 | 40 |
| 2022-07 | 383 | 39 | 38 | 39 | 38 | 40 |
| 2022-08 | 416 | 39 | 38 | 39 | 38 | 40 |
| 2022-09 | 351 | 39 | 38 | 39 | 38 | 40 |
| 2022-10 | 430 | 39 | 38 | 39 | 38 | 40 |
| 2022-11 | 416 | 39 | 38 | 39 | 38 | 40 |
| 2022-12 | 385 | 39 | 38 | 39 | 38 | 40 |
| 2023-01 | 370 | 39 | 38 | 39 | 38 | 40 |
| 2023-02 | 347 | 39 | 38 | 39 | 38 | 40 |
| 2023-03 | 371 | 39 | 38 | 39 | 38 | 40 |
| 2023-04 | 394 | 39 | 38 | 39 | 38 | 40 |

Supplementary Data 2(b): **NHS ‘Talking Therapies’**

| Month/year | Site A | | Site B | |
| --- | --- | --- | --- | --- |
|  | n NHS ‘Talking Therapies’ | Total | n NHS ‘Talking Therapies’ | Total |
| 2019-11 | 18 | 401 | 14 | 305 |
| 2019-12 | 13 | 396 | 15 | 313 |
| 2020-01 | 16 | 440 | 17 | 290 |
| 2020-02 | 20 | 402 | <10 | 274 |
| 2020-03 | 15 | 374 | 10 | 293 |
| 2020-04 | <10 | 374 | 11 | 255 |
| 2020-05 | 12 | 358 | 13 | 301 |
| 2020-06 | 13 | 391 | 12 | 270 |
| 2020-07 | 18 | 404 | 16 | 292 |
| 2020-08 | 16 | 390 | <10 | 247 |
| 2020-09 | 12 | 419 | 13 | 288 |
| 2020-10 | 10 | 389 | 20 | 289 |
| 2020-11 | 13 | 376 | 13 | 260 |
| 2020-12 | 14 | 338 | 11 | 213 |
| 2021-01 | 11 | 347 | 12 | 268 |
| 2021-02 | 17 | 361 | 14 | 266 |
| 2021-03 | 18 | 369 | 17 | 279 |
| 2021-04 | 15 | 393 | 12 | 247 |
| 2021-05 | <10 | 377 | 20 | 256 |
| 2021-06 | 11 | 373 | 14 | 266 |
| 2021-07 | 17 | 428 | 16 | 276 |
| 2021-08 | 12 | 402 | 15 | 268 |
| 2021-09 | 14 | 377 | 18 | 299 |
| 2021-10 | 18 | 425 | 24 | 292 |
| 2021-11 | 15 | 421 | <10 | 291 |
| 2021-12 | 24 | 393 | 17 | 261 |
| 2022-01 | 19 | 390 | 16 | 234 |
| 2022-02 | 16 | 362 | 17 | 218 |
| 2022-03 | 13 | 413 | <10 | 229 |
| 2022-04 | 17 | 386 | 20 | 260 |
| 2022-05 | 11 | 338 | 15 | 241 |
| 2022-06 | 387 | 774 | 11 | 242 |
| 2022-07 | 12 | 383 | 10 | 251 |
| 2022-08 | 23 | 416 | <10 | 222 |
| 2022-09 | 334 | 668 | 13 | 211 |
| 2022-10 | 20 | 430 | 16 | 259 |
| 2022-11 | 17 | 416 | 11 | 241 |
| 2022-12 | 16 | 385 | 19 | 244 |
| 2023-01 | <10 | 370 | 23 | 241 |
| 2023-02 | 16 | 347 | <10 | 230 |
| 2023-03 | 13 | 371 | 16 | 221 |
| 2023-04 | 22 | 394 | 15 | 220 |

Supplementary Data 2(c): **vaginal tear** (3rd or 4th degree)

| Month/year | Site A | | Site B | |
| --- | --- | --- | --- | --- |
|  | n vaginal tear | Total | n vaginal tear | Total |
| 2019-11 | <10 | 259 | <10 | 209 |
| 2019-12 | <10 | 250 | <10 | 223 |
| 2020-01 | <10 | 289 | <10 | 213 |
| 2020-02 | <10 | 252 | <10 | 189 |
| 2020-03 | <10 | 243 | <10 | 202 |
| 2020-04 | <10 | 247 | <10 | 167 |
| 2020-05 | <10 | 229 | <10 | 213 |
| 2020-06 | <10 | 256 | <10 | 167 |
| 2020-07 | <10 | 248 | <10 | 205 |
| 2020-08 | 11 | 269 | <10 | 172 |
| 2020-09 | <10 | 276 | <10 | 189 |
| 2020-10 | <10 | 262 | <10 | 199 |
| 2020-11 | <10 | 250 | <10 | 173 |
| 2020-12 | <10 | 227 | <10 | 148 |
| 2021-01 | <10 | 239 | <10 | 183 |
| 2021-02 | 12 | 242 | <10 | 166 |
| 2021-03 | <10 | 243 | <10 | 182 |
| 2021-04 | <10 | 253 | <10 | 147 |
| 2021-05 | <10 | 254 | <10 | 156 |
| 2021-06 | <10 | 229 | <10 | 160 |
| 2021-07 | <10 | 278 | <10 | 180 |
| 2021-08 | 13 | 263 | <10 | 162 |
| 2021-09 | <10 | 230 | <10 | 190 |
| 2021-10 | <10 | 274 | <10 | 187 |
| 2021-11 | <10 | 257 | <10 | 186 |
| 2021-12 | <10 | 228 | <10 | 167 |
| 2022-01 | <10 | 239 | <10 | 141 |
| 2022-02 | <10 | 200 | <10 | 132 |
| 2022-03 | <10 | 245 | <10 | 148 |
| 2022-04 | <10 | 201 | <10 | 153 |
| 2022-05 | <10 | 196 | <10 | 134 |
| 2022-06 | <10 | 218 | <10 | 124 |
| 2022-07 | <10 | 226 | <10 | 134 |
| 2022-08 | <10 | 234 | <10 | 141 |
| 2022-09 | <10 | 190 | <10 | 126 |
| 2022-10 | <10 | 246 | <10 | 163 |
| 2022-11 | <10 | 231 | <10 | 141 |
| 2022-12 | <10 | 196 | <10 | 142 |
| 2023-01 | <10 | 214 | <10 | 144 |
| 2023-02 | <10 | 186 | <10 | 130 |
| 2023-03 | <10 | 179 | <10 | 112 |
| 2023-04 | <10 | 213 | <10 | 119 |

Supplementary Data 2(d): **postpartum haemorrhage**

| Month/year | Site A | | Site B | |
| --- | --- | --- | --- | --- |
|  | N postpartum haemorrhage | Total | N postpartum haemorrhage | Total |
| 2019-11 | 40 | 401 | 18 | 305 |
| 2019-12 | 56 | 396 | 23 | 313 |
| 2020-01 | 52 | 440 | 23 | 290 |
| 2020-02 | 48 | 402 | 21 | 274 |
| 2020-03 | 56 | 374 | 19 | 293 |
| 2020-04 | 47 | 374 | 18 | 255 |
| 2020-05 | 32 | 358 | 20 | 301 |
| 2020-06 | 50 | 391 | 24 | 270 |
| 2020-07 | 36 | 404 | 22 | 292 |
| 2020-08 | 51 | 390 | 23 | 247 |
| 2020-09 | 41 | 419 | 13 | 288 |
| 2020-10 | 53 | 389 | 14 | 289 |
| 2020-11 | 43 | 376 | 21 | 260 |
| 2020-12 | 26 | 338 | 12 | 213 |
| 2021-01 | 35 | 347 | 25 | 268 |
| 2021-02 | 45 | 361 | 27 | 266 |
| 2021-03 | 42 | 369 | 19 | 279 |
| 2021-04 | 40 | 393 | 12 | 247 |
| 2021-05 | 42 | 377 | 16 | 256 |
| 2021-06 | 51 | 373 | 20 | 266 |
| 2021-07 | 56 | 428 | 27 | 276 |
| 2021-08 | 46 | 402 | 34 | 268 |
| 2021-09 | 47 | 377 | 29 | 299 |
| 2021-10 | 50 | 425 | 42 | 292 |
| 2021-11 | 57 | 421 | 28 | 291 |
| 2021-12 | 52 | 393 | 21 | 261 |
| 2022-01 | 40 | 390 | 16 | 234 |
| 2022-02 | 55 | 614 | 18 | 218 |
| 2022-03 | 55 | 413 | 21 | 229 |
| 2022-04 | 67 | 386 | 22 | 260 |
| 2022-05 | 33 | 338 | 26 | 241 |
| 2022-06 | 39 | 400 | 24 | 242 |
| 2022-07 | 40 | 383 | 21 | 251 |
| 2022-08 | 49 | 416 | <10 | 222 |
| 2022-09 | 35 | 351 | 13 | 211 |
| 2022-10 | 57 | 430 | 27 | 259 |
| 2022-11 | 52 | 416 | 16 | 241 |
| 2022-12 | 39 | 385 | 15 | 244 |
| 2023-01 | 45 | 370 | 21 | 240 |
| 2023-02 | 46 | 347 | 23 | 230 |
| 2023-03 | 37 | 371 | 20 | 221 |
| 2023-04 | 44 | 394 | 21 | 220 |
