# Supplementary material for: Temporal trends in pregnancy outcomes during a health system shock
Source: Commun Med (Lond). 2026 May 7;6:391. doi: 10.1038/s43856-026-01493-x (PMC13365597; doi:10.1038/s43856-026-01493-x)
Supplement: Supplementary file 5 — Supplementary Data 3 [file 43856_2026_1493_MOESM5_ESM.docx]

**SUPPLEMENTARY DATA 3**

Title: Source data for Figure 3

Legend: N/month outcomes for each of the following outcomes, for the total study population and for sites A and B separately: (a) emergency Caesarean; (b) elective Caesarean; (c) unassisted vaginal birth; and (d) assisted vaginal birth specifically. The N for outcomes with fewer than 10 events has been suppressed.

Supplementary Data 3(a): **emergency Caesarean**

| Month/year | Site A | | Site B | |
| --- | --- | --- | --- | --- |
|  | N emergency Caesareans | Total | N emergency Caesareans | Total |
| 2019-11 | 86 | 400 | 59 | 305 |
| 2019-12 | 97 | 396 | 59 | 312 |
| 2020-01 | 78 | 439 | 45 | 289 |
| 2020-02 | 82 | 402 | 51 | 274 |
| 2020-03 | 88 | 374 | 64 | 293 |
| 2020-04 | 73 | 374 | 46 | 252 |
| 2020-05 | 68 | 358 | 51 | 301 |
| 2020-06 | 82 | 390 | 64 | 269 |
| 2020-07 | 92 | 404 | 45 | 292 |
| 2020-08 | 75 | 390 | 43 | 246 |
| 2020-09 | 79 | 419 | 68 | 287 |
| 2020-10 | 80 | 389 | 57 | 288 |
| 2020-11 | 75 | 376 | 54 | 260 |
| 2020-12 | 67 | 337 | 43 | 213 |
| 2021-01 | 79 | 346 | 65 | 267 |
| 2021-02 | 71 | 359 | 62 | 265 |
| 2021-03 | 73 | 369 | 58 | 278 |
| 2021-04 | 85 | 392 | 63 | 247 |
| 2021-05 | 81 | 377 | 66 | 251 |
| 2021-06 | 81 | 373 | 62 | 263 |
| 2021-07 | 104 | 425 | 58 | 273 |
| 2021-08 | 95 | 402 | 57 | 268 |
| 2021-09 | 99 | 374 | 60 | 298 |
| 2021-10 | 81 | 425 | 57 | 289 |
| 2021-11 | 103 | 419 | 61 | 290 |
| 2021-12 | 95 | 393 | 52 | 261 |
| 2022-01 | 92 | 390 | 57 | 232 |
| 2022-02 | 109 | 360 | 50 | 215 |
| 2022-03 | 108 | 413 | 55 | 227 |
| 2022-04 | 121 | 386 | 68 | 258 |
| 2022-05 | 98 | 338 | 71 | 240 |
| 2022-06 | 110 | 398 | 74 | 241 |
| 2022-07 | 98 | 383 | 68 | 248 |
| 2022-08 | 114 | 415 | 52 | 222 |
| 2022-09 | 91 | 351 | 58 | 211 |
| 2022-10 | 98 | 430 | 65 | 257 |
| 2022-11 | 112 | 416 | 56 | 241 |
| 2022-12 | 111 | 382 | 63 | 242 |
| 2023-01 | 94 | 369 | 56 | 240 |
| 2023-02 | 97 | 347 | 52 | 227 |
| 2023-03 | 116 | 369 | 64 | 219 |
| 2023-04 | 121 | 394 | 60 | 219 |

Supplementary Data 3(b): **elective Caesarean**

| Month/year | Site A | | Site B | |
| --- | --- | --- | --- | --- |
|  | N elective Caesarean | Total | N elective Caesarean | Total |
| 2019-11 | 64 | 400 | 42 | 305 |
| 2019-12 | 68 | 396 | 37 | 312 |
| 2020-01 | 83 | 439 | 31 | 289 |
| 2020-02 | 66 | 402 | 40 | 274 |
| 2020-03 | 47 | 374 | 37 | 293 |
| 2020-04 | 50 | 374 | 44 | 252 |
| 2020-05 | 62 | 358 | 41 | 301 |
| 2020-06 | 62 | 390 | 42 | 269 |
| 2020-07 | 70 | 404 | 45 | 292 |
| 2020-08 | 56 | 390 | 36 | 246 |
| 2020-09 | 66 | 419 | 38 | 287 |
| 2020-10 | 64 | 389 | 38 | 288 |
| 2020-11 | 63 | 376 | 36 | 260 |
| 2020-12 | 51 | 337 | 24 | 213 |
| 2021-01 | 44 | 346 | 30 | 267 |
| 2021-02 | 68 | 359 | 46 | 265 |
| 2021-03 | 63 | 369 | 45 | 278 |
| 2021-04 | 68 | 392 | 42 | 247 |
| 2021-05 | 53 | 377 | 32 | 251 |
| 2021-06 | 80 | 373 | 43 | 263 |
| 2021-07 | 69 | 425 | 45 | 273 |
| 2021-08 | 57 | 402 | 45 | 268 |
| 2021-09 | 53 | 374 | 50 | 298 |
| 2021-10 | 82 | 425 | 46 | 289 |
| 2021-11 | 72 | 419 | 50 | 290 |
| 2021-12 | 72 | 393 | 44 | 261 |
| 2022-01 | 70 | 390 | 36 | 232 |
| 2022-02 | 65 | 360 | 41 | 215 |
| 2022-03 | 63 | 413 | 31 | 227 |
| 2022-04 | 74 | 386 | 36 | 258 |
| 2022-05 | 58 | 338 | 45 | 240 |
| 2022-06 | 72 | 398 | 50 | 241 |
| 2022-07 | 72 | 383 | 46 | 248 |
| 2022-08 | 79 | 415 | 29 | 222 |
| 2022-09 | 75 | 351 | 34 | 211 |
| 2022-10 | 87 | 430 | 38 | 257 |
| 2022-11 | 80 | 416 | 38 | 241 |
| 2022-12 | 70 | 382 | 41 | 242 |
| 2023-01 | 65 | 369 | 40 | 240 |
| 2023-02 | 57 | 347 | 38 | 227 |
| 2023-03 | 83 | 369 | 40 | 219 |
| 2023-04 | 70 | 394 | 39 | 219 |

Supplementary Data 3(c): **unassisted vaginal birth**

| Month/year | Site A | | Site B | |
| --- | --- | --- | --- | --- |
|  | n unassisted vaginal birth | Total | n unassisted vaginal birth | Total |
| 2019-11 | 186 | 400 | 154 | 305 |
| 2019-12 | 177 | 396 | 162 | 312 |
| 2020-01 | 212 | 439 | 165 | 289 |
| 2020-02 | 198 | 402 | 140 | 274 |
| 2020-03 | 182 | 374 | 139 | 293 |
| 2020-04 | 198 | 374 | 124 | 252 |
| 2020-05 | 178 | 358 | 155 | 301 |
| 2020-06 | 178 | 390 | 119 | 269 |
| 2020-07 | 184 | 404 | 156 | 292 |
| 2020-08 | 195 | 390 | 123 | 246 |
| 2020-09 | 201 | 419 | 144 | 287 |
| 2020-10 | 189 | 389 | 141 | 288 |
| 2020-11 | 177 | 376 | 122 | 260 |
| 2020-12 | 161 | 337 | 104 | 213 |
| 2021-01 | 151 | 346 | 116 | 267 |
| 2021-02 | 167 | 359 | 111 | 265 |
| 2021-03 | 174 | 369 | 132 | 278 |
| 2021-04 | 175 | 392 | 107 | 247 |
| 2021-05 | 182 | 377 | 109 | 251 |
| 2021-06 | 169 | 373 | 119 | 263 |
| 2021-07 | 191 | 425 | 127 | 273 |
| 2021-08 | 190 | 402 | 126 | 268 |
| 2021-09 | 163 | 374 | 141 | 298 |
| 2021-10 | 184 | 425 | 138 | 289 |
| 2021-11 | 174 | 419 | 131 | 290 |
| 2021-12 | 174 | 393 | 120 | 261 |
| 2022-01 | 178 | 390 | 103 | 232 |
| 2022-02 | 130 | 360 | 92 | 215 |
| 2022-03 | 180 | 310 | 100 | 227 |
| 2022-04 | 151 | 386 | 123 | 258 |
| 2022-05 | 149 | 338 | 88 | 240 |
| 2022-06 | 158 | 398 | 88 | 241 |
| 2022-07 | 150 | 383 | 98 | 248 |
| 2022-08 | 162 | 415 | 97 | 222 |
| 2022-09 | 145 | 351 | 92 | 211 |
| 2022-10 | 180 | 430 | 107 | 257 |
| 2022-11 | 171 | 416 | 115 | 241 |
| 2022-12 | 226 | 452 | 108 | 242 |
| 2023-01 | 163 | 369 | 106 | 240 |
| 2023-02 | 152 | 347 | 94 | 227 |
| 2023-03 | 137 | 369 | 84 | 219 |
| 2023-04 | 149 | 394 | 80 | 219 |

Supplementary Data 3(d): **assisted vaginal birth**

| Month/ year | Site A | | Site B | |
| --- | --- | --- | --- | --- |
|  | n assisted vaginal birth | Total | n assisted vaginal birth | Total |
| 2019-11 | 64 | 400 | 50 | 305 |
| 2019-12 | 54 | 396 | 54 | 312 |
| 2020-01 | 66 | 439 | 48 | 289 |
| 2020-02 | 56 | 402 | 43 | 274 |
| 2020-03 | 57 | 374 | 53 | 293 |
| 2020-04 | 53 | 374 | 38 | 252 |
| 2020-05 | 50 | 358 | 54 | 301 |
| 2020-06 | 68 | 390 | 44 | 269 |
| 2020-07 | 58 | 404 | 46 | 292 |
| 2020-08 | 64 | 390 | 44 | 246 |
| 2020-09 | 73 | 419 | 37 | 287 |
| 2020-10 | 56 | 389 | 52 | 288 |
| 2020-11 | 61 | 376 | 48 | 260 |
| 2020-12 | 58 | 337 | 42 | 213 |
| 2021-01 | 72 | 346 | 56 | 267 |
| 2021-02 | 53 | 359 | 46 | 265 |
| 2021-03 | 59 | 369 | 43 | 278 |
| 2021-04 | 64 | 392 | 35 | 247 |
| 2021-05 | 61 | 377 | 44 | 251 |
| 2021-06 | 43 | 373 | 39 | 263 |
| 2021-07 | 61 | 425 | 43 | 273 |
| 2021-08 | 60 | 402 | 40 | 268 |
| 2021-09 | 59 | 374 | 47 | 298 |
| 2021-10 | 78 | 425 | 48 | 289 |
| 2021-11 | 70 | 419 | 48 | 290 |
| 2021-12 | 52 | 393 | 45 | 261 |
| 2022-01 | 50 | 390 | 36 | 232 |
| 2022-02 | 56 | 360 | 32 | 215 |
| 2022-03 | 62 | 413 | 41 | 227 |
| 2022-04 | 40 | 386 | 31 | 258 |
| 2022-05 | 33 | 338 | 36 | 240 |
| 2022-06 | 58 | 398 | 29 | 241 |
| 2022-07 | 63 | 383 | 36 | 248 |
| 2022-08 | 60 | 415 | 44 | 222 |
| 2022-09 | 40 | 351 | 27 | 211 |
| 2022-10 | 65 | 430 | 47 | 257 |
| 2022-11 | 53 | 416 | 32 | 241 |
| 2022-12 | 45 | 382 | 30 | 242 |
| 2023-01 | 47 | 369 | 38 | 240 |
| 2023-02 | 41 | 347 | 43 | 227 |
| 2023-03 | 33 | 369 | 31 | 219 |
| 2023-04 | 54 | 394 | 40 | 219 |
