# Supplementary material for: Temporal trends in pregnancy outcomes during a health system shock
Source: Commun Med (Lond). 2026 May 7;6:391. doi: 10.1038/s43856-026-01493-x (PMC13365597; doi:10.1038/s43856-026-01493-x)
Supplement: Supplementary file 6 — Supllementary Data 4 [file 43856_2026_1493_MOESM6_ESM.docx]

**SUPPLEMENTARY DATA 4**

Title: Source data for Figure 4

Legend: N/month outcomes for each of the following outcomes, for the total study population and for sites A and B separately: (a) accessed secondary mental health services ‘Community contacts’; (b) labour induction; and (c) neonatal intensive care unit admission. The N for outcomes with fewer than 10 events has been suppressed.

Supplementary Data 4(a): **accessed secondary mental health services ‘Community contacts’**

| Month/year | Total population | | Site A | | Site B | |
| --- | --- | --- | --- | --- | --- | --- |
|  | n community contacts | Total | n community contacts | Total | n community contacts | Total |
| 2019-11 | 37 | 706 | 21 | 401 | 16 | 305 |
| 2019-12 | 31 | 709 | 14 | 396 | 17 | 313 |
| 2020-01 | 30 | 730 | 15 | 440 | 15 | 290 |
| 2020-02 | 20 | 676 | 10 | 402 | 10 | 274 |
| 2020-03 | 17 | 667 | <10 | 374 | 11 | 293 |
| 2020-04 | 17 | 629 | <10 | 374 | 10 | 255 |
| 2020-05 | 19 | 659 | <10 | 358 | 11 | 301 |
| 2020-06 | 21 | 661 | <10 | 391 | 12 | 270 |
| 2020-07 | 12 | 696 | <10 | 404 | <10 | 292 |
| 2020-08 | 11 | 637 | <10 | 390 | <10 | 247 |
| 2020-09 | 19 | 707 | 10 | 419 | <10 | 288 |
| 2020-10 | 21 | 678 | <10 | 389 | 13 | 289 |
| 2020-11 | 16 | 636 | 10 | 376 | <10 | 260 |
| 2020-12 | 15 | 551 | 13 | 338 | <10 | 213 |
| 2021-01 | 19 | 615 | <10 | 347 | 12 | 268 |
| 2021-02 | 22 | 627 | <10 | 361 | 15 | 266 |
| 2021-03 | 28 | 648 | 17 | 369 | 11 | 279 |
| 2021-04 | 15 | 640 | 11 | 393 | <10 | 247 |
| 2021-05 | 23 | 633 | 13 | 377 | 10 | 256 |
| 2021-06 | 23 | 639 | 14 | 373 | <10 | 266 |
| 2021-07 | 25 | 704 | 13 | 428 | 12 | 276 |
| 2021-08 | 28 | 670 | 18 | 402 | 10 | 268 |
| 2021-09 | 28 | 676 | 15 | 377 | 13 | 299 |
| 2021-10 | 31 | 717 | 13 | 425 | 18 | 292 |
| 2021-11 | 25 | 712 | 14 | 421 | 11 | 291 |
| 2021-12 | 28 | 654 | 13 | 393 | 15 | 261 |
| 2022-01 | 36 | 624 | 20 | 390 | 16 | 234 |
| 2022-02 | 21 | 580 | 11 | 362 | 10 | 218 |
| 2022-03 | 18 | 642 | 15 | 413 | <10 | 229 |
| 2022-04 | 29 | 646 | 13 | 386 | 16 | 260 |
| 2022-05 | 30 | 579 | 17 | 338 | 13 | 241 |
| 2022-06 | 27 | 642 | 17 | 400 | 10 | 242 |
| 2022-07 | 27 | 634 | 16 | 383 | 11 | 251 |
| 2022-08 | 35 | 638 | 20 | 416 | 15 | 222 |
| 2022-09 | 14 | 562 | <10 | 351 | <10 | 211 |
| 2022-10 | 33 | 689 | 21 | 430 | 12 | 259 |
| 2022-11 | 34 | 657 | 22 | 416 | 12 | 241 |
| 2022-12 | 23 | 629 | <10 | 385 | 14 | 244 |
| 2023-01 | 34 | 611 | 15 | 370 | 19 | 241 |
| 2023-02 | 24 | 577 | 17 | 347 | <10 | 230 |
| 2023-03 | 34 | 592 | 22 | 371 | 12 | 221 |
| 2023-04 | 26 | 614 | 16 | 394 | 10 | 220 |

Supplementary Data 4(b): **labour induction**

| Month/year | Site A | | Site B | |
| --- | --- | --- | --- | --- |
|  | n labour induction | Total | n labour induction | Total |
| 2019-11 | 214 | 400 | 151 | 305 |
| 2019-12 | 219 | 396 | 150 | 312 |
| 2020-01 | 227 | 439 | 124 | 289 |
| 2020-02 | 204 | 402 | 134 | 274 |
| 2020-03 | 192 | 374 | 154 | 293 |
| 2020-04 | 176 | 374 | 128 | 252 |
| 2020-05 | 180 | 358 | 146 | 301 |
| 2020-06 | 212 | 390 | 150 | 269 |
| 2020-07 | 220 | 404 | 136 | 292 |
| 2020-08 | 195 | 390 | 123 | 246 |
| 2020-09 | 218 | 419 | 143 | 287 |
| 2020-10 | 200 | 389 | 147 | 288 |
| 2020-11 | 199 | 376 | 138 | 260 |
| 2020-12 | 176 | 337 | 109 | 213 |
| 2021-01 | 195 | 346 | 151 | 267 |
| 2021-02 | 192 | 359 | 154 | 265 |
| 2021-03 | 195 | 369 | 146 | 278 |
| 2021-04 | 217 | 392 | 140 | 247 |
| 2021-05 | 195 | 377 | 142 | 251 |
| 2021-06 | 204 | 373 | 144 | 263 |
| 2021-07 | 234 | 425 | 146 | 273 |
| 2021-08 | 212 | 402 | 142 | 268 |
| 2021-09 | 211 | 374 | 157 | 298 |
| 2021-10 | 241 | 425 | 151 | 289 |
| 2021-11 | 245 | 419 | 159 | 290 |
| 2021-12 | 219 | 393 | 141 | 261 |
| 2022-01 | 212 | 390 | 129 | 232 |
| 2022-02 | 230 | 360 | 123 | 215 |
| 2022-03 | 233 | 413 | 127 | 227 |
| 2022-04 | 235 | 386 | 135 | 258 |
| 2022-05 | 189 | 338 | 152 | 240 |
| 2022-06 | 240 | 398 | 153 | 241 |
| 2022-07 | 233 | 383 | 150 | 248 |
| 2022-08 | 253 | 415 | 125 | 222 |
| 2022-09 | 206 | 351 | 119 | 211 |
| 2022-10 | 250 | 430 | 150 | 257 |
| 2022-11 | 245 | 416 | 126 | 241 |
| 2022-12 | 226 | 382 | 134 | 242 |
| 2023-01 | 206 | 369 | 134 | 240 |
| 2023-02 | 195 | 347 | 133 | 227 |
| 2023-03 | 232 | 369 | 135 | 219 |
| 2023-04 | 245 | 394 | 139 | 219 |

Supplementary Data 4(c): **Neonatal intensive care unit (NICU) admission**

| Month/year | Total population | | Site A | | Site B | |
| --- | --- | --- | --- | --- | --- | --- |
|  | n NICU admissions | Total | n NICU admissions | Total | n NICU admissions | Total |
| 2019-11 | 54 | 706 | 39 | 401 | 15 | 305 |
| 2019-12 | 57 | 709 | 41 | 396 | 16 | 313 |
| 2020-01 | 53 | 730 | 42 | 440 | 11 | 290 |
| 2020-02 | 49 | 676 | 38 | 402 | 11 | 274 |
| 2020-03 | 52 | 667 | 41 | 374 | 11 | 293 |
| 2020-04 | 31 | 629 | 22 | 374 | <10 | 255 |
| 2020-05 | 43 | 659 | 28 | 358 | 15 | 301 |
| 2020-06 | 49 | 661 | 35 | 391 | 14 | 270 |
| 2020-07 | 56 | 696 | 39 | 404 | 17 | 292 |
| 2020-08 | 40 | 637 | 31 | 390 | <10 | 247 |
| 2020-09 | 39 | 707 | 27 | 419 | 12 | 288 |
| 2020-10 | 45 | 678 | 35 | 389 | 10 | 289 |
| 2020-11 | 57 | 636 | 50 | 376 | <10 | 260 |
| 2020-12 | 47 | 551 | 41 | 338 | <10 | 213 |
| 2021-01 | 57 | 615 | 36 | 347 | 21 | 268 |
| 2021-02 | 54 | 627 | 35 | 361 | 19 | 266 |
| 2021-03 | 52 | 648 | 35 | 369 | 17 | 279 |
| 2021-04 | 46 | 640 | 32 | 393 | 14 | 247 |
| 2021-05 | 46 | 633 | 32 | 377 | 14 | 256 |
| 2021-06 | 43 | 639 | 33 | 373 | 10 | 266 |
| 2021-07 | 48 | 704 | 36 | 428 | 12 | 276 |
| 2021-08 | 51 | 670 | 39 | 402 | 12 | 268 |
| 2021-09 | 49 | 676 | 37 | 377 | 12 | 299 |
| 2021-10 | 25 | 717 | 15 | 425 | 10 | 292 |
| 2021-11 | 41 | 712 | 31 | 421 | 10 | 291 |
| 2021-12 | 34 | 654 | 19 | 393 | 15 | 261 |
| 2022-01 | 39 | 624 | 23 | 390 | 16 | 234 |
| 2022-02 | 36 | 580 | 25 | 362 | 11 | 218 |
| 2022-03 | 54 | 642 | 39 | 413 | 15 | 229 |
| 2022-04 | 43 | 646 | 31 | 386 | 12 | 260 |
| 2022-05 | 25 | 579 | 19 | 338 | <10 | 241 |
| 2022-06 | 27 | 642 | 19 | 400 | <10 | 242 |
| 2022-07 | 38 | 634 | 20 | 383 | 18 | 251 |
| 2022-08 | 21 | 638 | 18 | 416 | <10 | 222 |
| 2022-09 | 36 | 562 | 27 | 351 | <10 | 211 |
| 2022-10 | 40 | 689 | 26 | 430 | 14 | 259 |
| 2022-11 | 38 | 657 | 30 | 416 | <10 | 241 |
| 2022-12 | 38 | 629 | 22 | 385 | 16 | 244 |
| 2023-01 | 38 | 611 | 29 | 370 | <10 | 241 |
| 2023-02 | 13 | 577 | <10 | 347 | <10 | 230 |
| 2023-03 | 28 | 592 | 19 | 371 | <10 | 221 |
| 2023-04 | 29 | 614 | 13 | 394 | 16 | 220 |
